# Applications of laboratory findings in the prevention, diagnosis, treatment, and monitoring of COVID-19

**DOI:** 10.1038/s41392-021-00731-z

**Published:** 2021-08-25

**Authors:** Zirui Meng, Shuo Guo, Yanbing Zhou, Mengjiao Li, Minjin Wang, Binwu Ying

**Affiliations:** Department of Laboratory Medicine, West China Hospital, Sichuan University, Chengdu, Sichuan Province China

**Keywords:** Infectious diseases, Predictive markers

## Abstract

The worldwide pandemic of coronavirus disease 2019 (COVID-19) presents us with a serious public health crisis. To combat the virus and slow its spread, wider testing is essential. There is a need for more sensitive, specific, and convenient detection methods of the severe acute respiratory syndrome coronavirus 2 (SARS-CoV-2). Advanced detection can greatly improve the ability and accuracy of the clinical diagnosis of COVID-19, which is conducive to the early suitable treatment and supports precise prophylaxis. In this article, we combine and present the latest laboratory diagnostic technologies and methods for SARS-CoV-2 to identify the technical characteristics, considerations, biosafety requirements, common problems with testing and interpretation of results, and coping strategies of commonly used testing methods. We highlight the gaps in current diagnostic capacity and propose potential solutions to provide cutting-edge technical support to achieve a more precise diagnosis, treatment, and prevention of COVID-19 and to overcome the difficulties with the normalization of epidemic prevention and control.

## Background

**Fig. 1:**
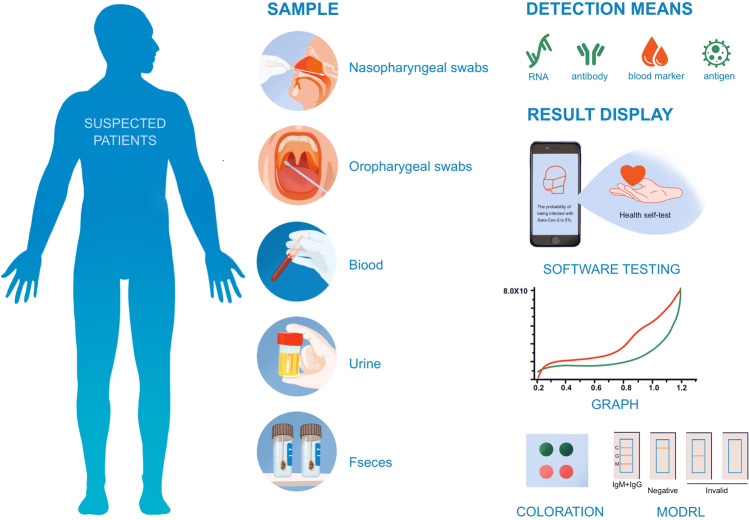
Laboratory diagnostic method of COVID-19

The global pandemic of the coronavirus disease 2019 (COVID-19)^1^ has threatened tens of thousands of people’s lives^2–4^ since its spread in 2019. It has a continuous negative effect on human health, economic growth, social stability, and eventually the civilization process of human society. At the same time, it emphasizes the importance of timely recognition, monitoring, prevention, management, and urgent intervention.^5–11^ While there is still no vaccine that can provide absolute protection, it is of great significance to develop swift and reliable diagnostic methods (Fig. 1) for the diagnosis of symptomatic or asymptomatic COVID-19 cases^12^; such well-based diagnostics are the key to quick and reliable treatment decisions and quarantine strategies,^13^ which can slow down the spread of this infectious disease.^14^ To identify COVID-19 infection, some conventional testing methodologies like thoracic imaging, computed tomography (CT) scan,^15,16^ portable chest X-ray,^17^ and flexible bronchoscopy^18^ have been used as supplement tools, while the quantitative real-time reverse-transcription-polymerase chain reaction (rRT-PCR) is currently regarded as the most popular test.^19^ However, limited by the unsatisfactory sensitivity on samples with a low virus load, advanced technologies (ddPCR,^20^ LAMP, RPA,^21^ CRISPR-Cas,^22^ and nanotechnology-based biosensors^23^) and big data analysis based on artificial intelligence^24^ are being investigated. Developing advanced, rapid, and timely diagnostic methods is a necessary complement to overcome the limitations of traditional techniques and will greatly strengthen our capabilities to defeat the epidemic.

Although this issue represents an important topic, the existing works on diagnostic tools are mostly partial and focus on different areas, e.g., nucleic acids, serological tests, new materials, or artificial intelligence. In this work, we intend to provide a comprehensive illustration of existing detection methods from a laboratory perspective, highlighting gaps in current diagnostic capacity and proposing potential solutions, rather than reiterating all the details included in previous publications. Herein, we review and summarize several COVID-19 detection technologies, along with their advantages and disadvantages. Besides, the technical characteristics, considerations, biosafety requirements, common problems with testing and interpretation of results, and coping strategies of commonly used testing methods are discussed to provide cutting-edge technical support to achieve more precise diagnosis treatment and prevention of COVID-19 and to overcome the difficulties with the normalization of epidemic prevention and control.

## Pathogen-based laboratory findings for COVID-19 detection

### Nucleic acid amplification testing

**Fig. 2:**
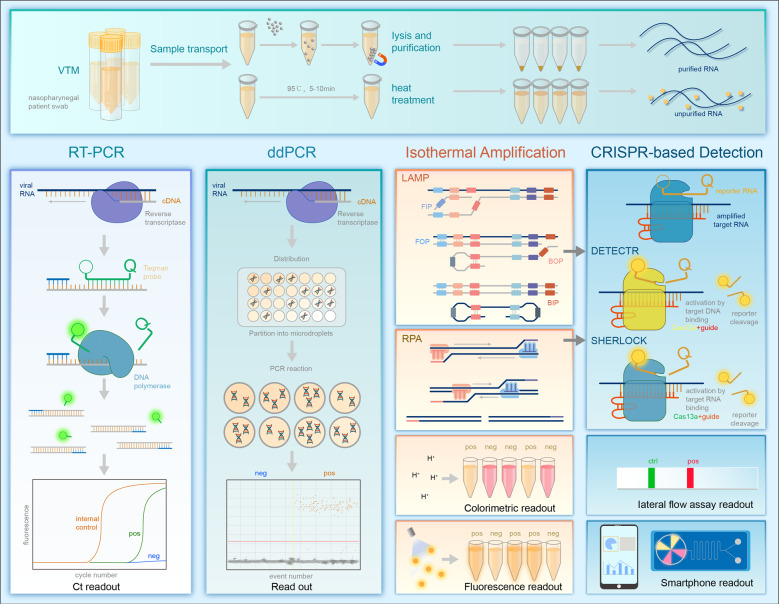
Summary of the nucleic acid amplification testing. The nucleic acid of SARS-CoV-2 is extracted by magnetic beads absorption or heat treatment. Amplification is performed and signals could be detected using the special instrument for rRT-PCR, ddPCR, LAMP, and RPA. Isothermal amplification and CRISPR-based methods can be read out by a color change, lateral-flow assay, fluorescence signal or portable electronic devices

With the rapid global spread of severe acute respiratory syndrome coronavirus 2 (SARS-CoV-2), COVID-19 is threatening public health. Clinically, SARS-CoV-2 infection presents with highly heterogenic manifestations, ranging from asymptomatic infection to severe disease. The use of early nucleic acid amplification testing will help to discover and isolate new infection cases, thus limiting the SARS-CoV-2 transmission in public and improving the treatment outcome (Fig. 2).^25^ Here, we summarized the characteristics of several methods for SARS-CoV-2 detection (Table 1).

**Table 1:** Summary of nucleic acid amplification testing for SARS-CoV-2

Principle	Manufacturer	Target gene	Mock sample	Clinical sample	Sensitivity	Specificity	Limit of detection	Turn-around time	Reference
rRT-PCR	Laboratory-developed method	*RdRp, E* HKU-*N, ORF1* CCDC-*N, ORF1* *N1, N2, N3*	24	/	100%	/	500 copies/reaction	/	^29^
rRT-PCR	13 Commercial PCR kits	*RdRp, E* *ORF1ab, S, N*	3	/	/	/	3.3–330 copies	/	^30^
rRT-PCR	Luminex Molecular Diagnostics	*E*	/	214	97.8%	100%	200 copies/ml	4.5–5 h	^31^
rRT-PCR	Cepheid	*E, N2*	22	481	99.5%	95.8%	0.01PFU/ml	45 min	^32^
ddPCR	Bio-Rad	*RdRp, E* HKU-*N, ORF1* CCDC-*N, ORF1* *N1, N2, N3*	10	/	/	/	1–3 copies/reaction	/	^49^
ddPCR	Bio-Rad	*CCDC-N, ORF1*	/	77	94%	100%	1.8–2.1 copies/reaction	/	^50^
RT-LAMP	Laboratory-developed method	*ORF1ab*	/	248	89.9%	/	/	/	^58^
RT-LAMP	Laboratory-developed method	*Orf1a*	40	/	/	/	1 copy/ml	30 min	^56^
RT-LAMP	Laboratory-developed method	*ORF1ab, N*	/	129	100%	100%	12 copies/reaction	1 h	^60^
RT-LAMP	Laboratory-developed method	*N*	/	10	100%	100%	50 copies/µl	30 min	^61^
RT-RPA	Laboratory-developed method	*ORF1ab*	/	947	97.63%	97.87%	2 copies/reaction	15 min	^64^
RT-RPA	Laboratory-developed method	*N, S*	/	51	/	/	5 copies/	45 min	^65^
CRISPR-Cas13a-based SHERLOCK	Laboratory-developed method	*S, N, ORF1ab*	/	534	96.3%	100%	42 copies/reaction	/	^74^
CRISPR-Cas12a-based DETECTR	Laboratory-developed method	*N2, S*	/	60	95%	100%	10 copies/µl	45 min	^75^
AIOD-CRISPR assay	Laboratory-developed method	*N*	/	28	100%	100%	3 copies	40 min	^76^
Electric field and CRISPR-based diagnostics	Laboratory-developed method	*N, E*	/	64	30/32	30/30	10 copies/ml	40 min	^80^

#### Real-time reverse-transcription-polymerase chain reaction (rRT-PCR)

The rRT-PCR method was recommended for the detection of SARS-CoV-2 by the World Health Organization (WHO) and the Centers for Disease Control and Prevention (CDC).^25–27^ Several primer–probe sets were globally designed and used for the detection of SARS-CoV-2, including *N* and *ORF1-nsp10* (China CDC), *N1*, *N2* and *N3* (United States CDC), *N* and *ORF1-nsp14* (Hong Kong University), and *E*, *N* and *RdRp* (Charité Institute of Virology, Universitätsmedizin Berlin, Germany).^25–28^ These primer–probe sets were independently evaluated using rRT-PCR assays and shown to have a sensitivity of 100% for the detection of SARS-CoV-2 at 500 copies per reaction in mock clinical samples. However, this was not the case for the *RdRp* gene (Charité),^29^ which is not a reliable tool to detect SARS-CoV-2 due to the mismatches in the primer and probe binding regions. Specifically, nucleotide substitutions occurred at genome position 15519 during SARS-CoV-2 transmission, which altered the sensitivity of the *RdRp* (Charité) PCR assay.^29^

Based on the above-mentioned designed primer–probe sets, a series of commercial kits were developed for the detection of SARS-CoV-2 in clinical samples. These commercial kits were shown to have a PCT efficiency of >90% according to the study of Iglói et al., in which the performance characteristics of 13 commercial RT-PCR assays were assessed.^30^ Regarding the analytical sensitivity, it slightly varied between different commercial kits, such that the lowest amount of RNA copies detectable in 3/3 replicates varied from 3.3 RNA copies to 330 RNA copies.

Another important aspect to evaluate the commercial kits is the time required for SARS-CoV-2 detection. The average time of Luminex NxTAG CoV extended panel was around 4.5–5 h.^31^ However, the Cepheid Xpert Xpress system had a short time with approximately 45 min.^32^ To further simplify the process flow, heating-inactivation (95 °C, 5 min or 10 min) or adding lysate (Triton-X-100 or Tween-20) to the samples was conducted in clinical samples, and direct RT-PCR was performed on the RNA extraction-free samples.^33,34^ It is worth mentioning that the sensitivity of direct RT-PCR is slightly lower than traditional assays with 4 Ct value delay; this is because the genome RNA might be broken into shorter fragments, leading to poor detection performance.^34^

The positive detection rate of rRT-PCR assays has been recently reported to reach 89% at 0–4 days after symptom onset, dropping to 54% after 10–14 days.^35^ For the viral shedding, it may begin 5–6 days before the appearance of symptoms and can last up to 37 days in survivors,^36,37^ even longer in certain immunocompromised patients with diabetes mellitus or malignant tumors, due to the weak immune response and virus clearance.^38–40^ It is difficult to diagnose patients with low viral load using rRT-PCR assays at the early stage and rehabilitation phase of COVID-19.^41–43^ As a result, false-negative results are inevitable since viral RNA contents were lower than the detection limit, and a more sensitive method needs to be established to be used in such cases.^35,44^

#### Droplet digital polymerase chain reaction (ddPCR)

The method of droplet digital PCR (ddPCR) is a novel approach to perform absolute target nucleic acid quantification without the need for a standard curve. Using the same primers and probes as rRT-PCR, ddPCR has achieved improved sensitivity and precision for the detection of low viral load.^45–47^ Each microdroplet contains zero or one copy of the target fragment, which consists of thousands of micro PCR reactors. Based on Poisson statistics, the number of DNA molecules in the original sample was directly calculated, which reduces the subjectivity of the analysis by eliminating the need for signal threshold determination and standard curves.^45,48^

For emerging infectious diseases, like SARS-CoV-2 virus, ddPCR represents a promising approach for causative agent detection in patients with low viral load. The performance of rRT-PCR and that of ddPCR have been recently compared with the same samples using 8 primer–probe sets (United States CDC *N1, N2*, and *N3*; China CDC *ORF1* and *N*, HKU *N* and *ORF1*; Charité, *E*).^49^ The results demonstrated that ddPCR performed well in distinguishing positive cases from low viral load specimens (10^−4^ dilution) regardless of the used primer–probe set.^49^ The reportable range of the two methods has also been explored by another study using the *ORF1ab* and *N* genes as follows: 10–5 × 10^4^ copies/reaction for ddPCR and 1000–10^7^ copies/reaction for rRT-PCR.^50^ When the Ct value was higher than 34, the results of rRT-PCR and ddPCR were highly inconsistent. However, the ddPCR method showed better performance compared with routine rRT-PCR in the clinical samples with low virus load.^51^

Although it requires skilled technicians and special equipment, ddPCR represents an ideal method for the medical management of COVID-19. The method assists in discovering new cases with low viral load and keeping close and general contacts in quarantine at an early stage, thus achieving timely prevention of human-to-human transmission. Moreover, changes in the virus copy number provide evidence for evaluating the treatment effect and virus clearance rate and continuously monitoring the viral load in convalescent patients, which helps in policymaking and management of isolated patients.

#### Isothermal amplification

Multiple isothermal amplification assays were developed for pathogen identification, including loop-mediated isothermal amplification (LAMP), recombinase polymerase amplification (RPA), nucleic acid sequence-based amplification (NASBA), helicase-dependent amplification (HDA), strand-displacement amplification (SDA), and rolling circle amplification (RCA).^52^ Using these techniques, nucleic acid amplification testing could be performed at a constant temperature without a thermal cycler, which contributes to achieving point-of-care testing and improving public health in underdeveloped regions.^53–55^ Specifically, LAMP and RPA were widely used for emerging infectious pathogen detection, like the SARS-CoV-2 virus.

##### Loop-mediated isothermal amplification (LAMP)

In an LAMP experiment, four to six primers are specially designed to identify six to eight regions of the target sequence.^56,57^ The amplification begins with an inner primer invasion, then a strand-displacing polymerase elongates the primer and separates the DNA duplex. Subsequently, the first product is displaced by another strand, which is extended by an outer primer. Next, a dumbbell structure is formed and seeded to perform the exponential lamp amplification at a constant temperature. Microgram quantities of DNA were produced in such a fast reaction, and the results can be read out in an hour using turbidity, fluorescence dye, pH indicator, lateral-flow biosensor, or electronic devices.^56^

For RNA detection, as in the case of the SARS-CoV-2 virus, a reverse-transcription step was merged into the LAMP protocol, resulting in a reverse-transcription loop-mediated isothermal amplification (RT-LAMP). An RT-LAMP protocol for the rapid detection of SARS-CoV-2 was established by Yu et al., which indicated the specific amplification of *ORF1ab* by a color change from pink to yellow as observed with the naked eye.^58^ The method was validated by 248 clinical samples and showed a sensitivity of 89.9% (223/248), and samples with a Ct value >37 showed a false-negative result, which indicates that RT-LAMP did not perform well in low viral load samples.^58^ To improve the detection performance, a variety of methods have been tested to enhance the testing efficiencies.

A series of additives and compounds with RNA protection properties were screened, and it was found that 40–50 mM guanidine chloride can dramatically improve the amplification speed while achieving a five- to tenfold increase in sensitivity.^59^ Furthermore, Rabe et al. added tris(2-carboxyethyl) phosphine (TCEP) and the divalent cation chelator ethylenediaminetetraacetic acid (EDTA) to the sample to inactivate the virions and purify viral RNA.^56^ While the former inhibited the formation of disulfide bridges, the latter inactivated endogenous RNases during the process of heat treatment (95 °C for 5 min) without RNA extraction.^56^ Combined with the RT-LAMP assay, the use of this protocol achieved a detection sensitivity of 1 copy per microliter.^56^

In addition, nanoparticle-based lateral-flow assays (LFAs) provided a new readout for RT-LAMP assays, which assisted in avoiding the potential subjective interpretation of the results.^60^ Based on the antigen–antibody and biotin/treptavidin interactions, FITC-/digoxin- and biotin-labeled duplex amplicons were simultaneously detected using LFB, coated with anti-FITC/digoxin and treptavidin. The final specific crimson band indicated the amplification of the *ORF1ab* or *N* genes and the limit of detection (LoD) was 12 copies/reaction.^60^ The validation using clinical oropharynx swab samples demonstrated a sensitivity of 100% (33/33, confirmed COVID-19 cases) and a specificity of 100% (96/96, non-COVID-19 cases).^60^

Besides LFA, a real-time optical fluorescence image could be acquired using a smartphone without opening a reaction chamber.^61^ It has been demonstrated that the *N* primer had the lowest LoD with 50 copies/microliter, compared with the *Orf1a*, *Orf8*, and *S* primers. The point-of-care testing system was evaluated by ten clinical samples and the acquired results were completely consistent with the RT-PCR assays.^61^

##### Recombinase polymerase amplification (RPA)

Recombinase polymerase amplification (RPA) or recombinase-aided amplification (RAA) is an isothermal amplification method, which achieves amplification using a combination of recombinase, single-strand DNA binding protein, strand-displacement DNA polymerase, and specific primers.^62^ It is faster than other isothermal amplification methods and can be performed at a constant temperature (37–42 °C).^63^

In order to timely discover and curb the transmission of the emerging pathogen of SARS-CoV-2, reverse-transcription RPA (RT-RPA) was developed for the rapid detection of the pathogen in the clinical laboratory. Wang et al. conducted an RT-RPA experiment for the detection of SARS-CoV-2 using a portable device at 39 °C.^64^ After adding extracted RNA, only 15 min were needed to obtain the result. Furthermore, the method was evaluated with the use of 947 clinical samples and achieved sensitivity and specificity of 97.63% (330/338) and 97.87% (596/609), respectively.^64^ In addition, an enhanced RT-PRA assay was established for the detection of SARS-CoV-2 by adding RNase H, selectively degrading the RNA strand in the RNA–DNA hybrid duplex.^65^ Combined with a commercial lateral-flow assay, the enhanced RT-PRA assays have taken only 45 min from collecting the sample to obtaining the result without RNA extraction.^65^ Its sensitivity was significantly improved and the LoD reached five viral copies with a minimal device.^65^ However, when the Ct value was higher than 32, the enhanced RT-RPA assay did not achieve a good performance and could not distinguish weak positive signals from the samples, especially for unextracted ones.^65^

Although the sensitivity of isothermal amplification methods, like RT-LAMP and RT-RPA, is slightly lower than rRT-PCR in low viral load samples, they have the potential for low-cost point-of-care testing. In some places like the airport, emergency department, and seafood market, portable devices can be set to achieve rapid SARS-CoV-2 detection, monitor, the virus contamination of the environment, and assist in blocking the virus transmission.

#### Clustered regularly interspaced short palindromic repeats (CRISPRs) and CRISPR-associated proteins (CRISPR-Cas)

The CRISPR-Cas system was initially discovered in prokaryotes and shown to play a vital role in protecting the organisms from the exogenous nucleic acid.^66^ It consists of 6 types and 22 subtypes and has genome editing properties and cleaving abilities.^66^ Among the subtypes, Cas13a is an RNA-guided, RNA-targeting enzyme, while Cas12a is an RNA-guided, DNA-targeting enzyme. They both have cleavage activity and are commonly used for pathogen detection.^66,67^ Combined with isothermal amplification, the specific high-sensitivity enzymatic reporter unlocking (SHERLOCK) platform based on CRISPR-Cas13a and DNA endonuclease-targeted CRISPR trans reporter (DETECTR) platform based on CRISPR-Cas12a have been explored for the detection of infectious pathogens, like the human papillomavirus, Dengue, and Zika viruses, since it was described in 2018.^68–73^

Since the COVID-19 outbreak, SHERLOCK and DETECTR platforms have been applied for the detection of SARS-CoV-2. Patchsung et al. performed a two-step CRISPR-Cas13a-based SHERLOCK experiment for the detection of SARS-CoV-2 viral RNA.^74^ Specific primer pairs and guide RNA sequences were designed and tested for the *S*, *N*, and *ORF1ab* genes. RT-RPA was applied to amplify the target sequences of the viral genome, followed by the identification and detection of specific sequences based on CRISPR-Cas13a. For the final results readout, fluorescence and lateral-flow assays were used.^74^ The results showed that the detection of the *S* gene was more sensitive than the *N* and *ORF1ab* genes with an LoD of 42 copies/reaction. These results were then clinically validated using a total of 534 clinical samples. The corresponding results illustrated that the SHERLOCK fluorescence readout performed better than the SHERLOCK lateral-flow readout, with a sensitivity of 96.3% and a specificity of 100%.^74^ Simultaneously, Broughton et al. developed and validated a CRISPR-Cas12a-based DETECTR platform for the detection of the SARS-CoV-2 virus.^75^ The fragments of the *N2* and *S* genes were isothermally amplified using the RT-LAMP assay, followed by a Cas12a-based specific sequence cleavage. Then, the FAM-biotin-labeled reporter was released and the signal could be visualized on a lateral-flow strip or read out using a fluorescent plate reader.^75^ The estimated LoD of the DETECTR assay was ten copies/μL reaction. Out of the 60 clinical samples (30 positive and 30 negative samples confirmed by rRT-PCR), the positive and negative predictive agreements were 95% and 100%, respectively.^75^

One problem regarding both SHERLOCK and DETECTR assays is that they have the potential risk to contaminate the laboratory with aerosol production when the reaction tube lid gets on. In order to avoid this problem, an All-In-One Dual CRISPR-Cas12a (AIOD-CRISPR) assay was established for one-step detection of SARS-CoV-2.^76^ Specific Cas12a-guide RNA complexes bound with the regions that were located near the identification sites of the primers in the target *N*-gene. When the reaction tube was incubated at 37 °C, the RT-RPA amplifications were triggered, and the products were identified and cleaved by the Cas12a-guide RNA complexes.^76^ Consequently, a single-stranded DNA fluorophore-quencher (ssDNA-FQ) reporter was released for a fluorescence-based readout. The AIOD-CRISPR assay achieved an ultrasensitive detection in one-pot reaction with an LoD of three copies. A total of 28 clinical swab samples were detected using purified RNA. The final results were completely in consistence with those obtained by the rRT-PCR method.^76^ Interestingly, another one-step method was also explored for viral RNA detection.^77–79^ Viral RNA templates and a reaction mixture of the RT-LAMP assay were added to the bottom of a tube and covered with mineral oil. At the same time, CRISPR-Cas12a reagents were pre-added inside the lid of the reaction tube.^77–79^ After isothermal amplification, the CRISPR-Cas12a reagents were mixed with the reaction solution by handshaking.^77–79^ Thus, target fragments could be identified, while avoiding potential contamination of the products.

Moreover, the CRISPR-based methods have also been combined with the microfluid technology to simplify the experimental process. Ramachandran et al. applied an electrokinetic microfluidic technique, called the isotachophoresis (ITP), to perform an automatic viral RNA extraction from raw samples and activate the cleavage abilities of CRISPR-Cas12a.^80^ The method of ITP utilized a two-buffer system, which included a high-mobility leading electrolyte (LE) buffer and a low-mobility trailing electrolyte (TE) one. Driven by electronic field, the sample ions selectively moved to the zone at the LE-to-TE interface.^80^ As a result, the nucleic acid purification, reagent mixing, and reaction acceleration could easily be achieved on a microfluid chip. The method dramatically improved the detection efficiency, taking only 30–40 min from the raw sample to answer.^80^

Combined with the isothermal amplification, microfluid system, lateral-flow assay, and fluorescence-based readout, the CRISPR-Cas-based methods could be used for point-of-care testing. Compared with single isothermal amplification methods, methods based on CRISPR-Cas have a higher specificity because the guide RNA could identify target sequences.

#### Genome sequencing

Advanced sequencing techniques, such as next-generation sequencing (NGS) and nanopore sequencing, have a pivotal role in emerging pathogen identification and real-time tracking.^81,82^ At the beginning of the SARS-CoV-2 outbreak, Zhou et al. collected bronchoalveolar lavage fluid from a critically ill patients and performed metagenomic analysis using NGS for pathogen identification.^83^ A 29891-base-pair genome of the potential causative agent was acquired, and the phylogenetic analysis demonstrated that the pathogen sequence was 96% identical to a bat coronavirus and 79.6% identical to the severe acute respiratory syndrome coronavirus (SARS-CoV).^83^ This led to the successful identification of the whole-genome sequence of the SARS-CoV-2 virus and paved the way to develop PCR-based methods and explore the potential pathogenic mechanism.

With the rapid spread of SARS-CoV-2, genome sequencing was commonly used for the molecular epidemiological surveillance and virus strain traceability analysis.^84,85^ The surveillance of emerging variants contributed to the discovery of strains able to spread easier,^86^ escape common rRT-PCR detection,^87^ cause more severe symptoms and evade natural immunity or vaccine-induced acquired immunity.^88^

It has been reported by numerous studies that the strain carrying a transversion mutant A23403G had become predominant in Europe, Oceania, South America, and Africa.^89^ The mutant strain caused a D614G (aspartate to glycine in protein position 614) change of the Spike protein.^89^ Indeed, in vitro engineered experiments demonstrated that the D614G substitution contributed to the viral replication and enhanced the virus infectivity and stability of virions in the human lung epithelial cells and primary human airway tissues.^90^ Furthermore, a new variant with an N501Y mutation emerged in the United Kingdom in the fall of 2020 and rapidly became the predominant strain in late November. Until December 2020, the strain had already been identified in 21 countries and regions, including America, Denmark, Italy, Japan, Spain, Singapore, and the United States.^91,92^ Structural biological analysis indicated that the N501Y mutant might increase the binding of the human angiotensin-converting enzyme 2. As a result, its transmission is easier than other mutants and a mandatory quarantine is necessary to suppress its spreading.^91^ More studies need to be done to explore the biological effects of the mutants in the future.

Epidemiological surveillance can help to discover the SARS-CoV-2 infection cluster events and contribute to the identification of the infection source.^93,94^ In the work of Lemieux et al., 772 complete genomes of SARS-CoV-2 in the Boston area at the early stage of the pandemic were analyzed.^94^ The results revealed two superspreading events: one happened in a skilled nursing facility and another at an international conference, which caused extensive international transmission.^94^

With the rapid spread of SARS-CoV-2, genomic surveillance will be continuously performed to discover new mutants, which will have a critical effect on the COVID-19 precaution, treatment, and vaccine-induced immunity.

### Serological testing

Due to asymptomatic infections or limited detection capacity,^95,96^ not all COVID-19 patients can get direct evidence of infection. Therefore, serological tests based on the detection of specific SARS-CoV-2 antibodies are important means for auxiliary purposes.^97,98^ Besides, due to the dynamic change in the levels of antibodies against SARS-CoV-2 during the different periods of infection, serological antibody detection plays a major role in previous infection identification, convalescent diagnosis, epidemiological investigation, and vaccine-effect evaluation.

#### Specific antibody profiles for serological testing

It is essential to understand the specific antibody profiles for identifying COVID-19, predicting the disease severity, and assessing long-term immune function. Some studies have shown that the median seroconversion time for IgM was 10–12 days after symptom onset, and it could be detected within 1 week in some of the patients.^98–102^ It increased at 2–3 weeks, reaching or getting close to the peak state, and then began to significantly decrease after 4–5 weeks, which was maintained for a short time in vivo.^103–107^ IgA was shown to be similar to IgM and produced earlier.^108,109^ The median seroconversion time was about 11 days and reached a peak at 3 weeks, maintaining a high level until about 6 weeks, with the positive rate close to 100%.^109–113^ Specific IgG generally appeared later than IgM, and the median time of seroconversion was 12–14 days.^97,98,104,105,109^ However, in Long’ study, the seroconversion time of IgG was earlier than that of IgM.^114^ Subsequently, it rose rapidly and reached a peak level at 3–4 weeks with a positive rate of 80.0–100.0%, which could generally be maintained until the eighth week.^105,107,115–119^ Besides, except for a few severe patients in which no neutralizing antibodies could be detected even at 3 weeks after onset, most patients could produce neutralizing antibodies within 1–4 days, with a low antibody level though.^110,120–122^ It significantly increased from the second week to reach a peak at 5–6 weeks, and then maintained a stable state or slightly decreased, such that the decrease in symptomatic patients was more obvious than that in asymptomatic patients.^122–125^ It has been recently observed that some antibody dynamic levels are still controversial, which is closely related to the severity and presence of symptoms.^123,126–128^

#### Enzyme-linked immunosorbent assay (ELISA) and chemiluminescence immunoassay (CLIA) used for SARS-CoV-2 serological detection

ELISA is an old universal chemical test widely available in most laboratories.^129–131^ Specific antigens, such as S1 domain, receptor-binding domain (RBD), S2 domain of spike protein, and nucleocapsid protein, against antibodies including IgM, IgG, and IgA generated due to SARS-CoV-2 are coated on the surface of the solid-phase carrier, and enzyme-labeled anti-IgM, anti-IgG or anti-IgA are added to bind with specific antibodies. Relevant substrates would react with the label enzyme and induce the color change, which can be detected using spectrophotometry to realize the qualitative or semi-quantitative detection of antibodies. The early ELISA kit available on the market was the Wantai SARS-CoV-2 Ab ELISA kit developed by the Beijing Wantai Biological Pharmacy company to capture the total antibody against SARS-CoV-2, which achieved the specificity of 97.5% and sensitivity of 96.7%.^132^ The subsequent ELISA kits from Bio-Rad Labs and Mount Sinai Hospital Clinical Laboratory for total antibody detection increased the specificity to 99.6% and 100%, respectively. However, the sensitivity remains at a relatively lower level of 92.2% and 92.5%,^133,134^ respectively. Compared with the total antibody detection, the test of separate specific antibodies including IgM, IgG, or IgA alone remains the major analyte of interest, which can also achieve better performance. The IgM and IgG are primarily tested because IgM is produced first at the early stage of infection and IgG has the highest production with long duration, suggesting the middle and late stage of infection or previous infection.^135–137^

In addition to the ELISA assays, other commonly used immunological methods include the chemiluminescence immunoassay (CLIA), electrochemiluminescence immunoassay (ECLIA), and enzyme-linked fluorescent assay (ELFA), some of which are also approved and available on the market (Supplementary Table S1). Most of these methods have gradually been developed into automatized assays, allowing hands-free processing and fast, high-throughput analysis. The CLIA/ECLIA methods are universally utilized technology integrated on automated equipment. They combine the highly sensitive chemiluminescence assay technology and highly specific immune reaction. This method has a higher sensitivity than that of ELISA, high specificity, wide linear range, and stable results. The CLIA/ECLIA assays launched out by companies like Abbott, Roche Diagnostics, Siemens Healthcare Diagnostic Inc and others can achieve both high sensitivity and specificity of more than 99% according to their reports.^138–140^ A study has been carried out to compare sixteen serological SARS-CoV-2 immunoassays in sixteen clinical laboratories in Denmark. The results revealed that the performance of most total-Ab and IgG assays, including ELISA, CLIA, and ECLIA with feasibility for high-throughput processing on automated platforms, reached acceptable criteria.^141^ Similarly, a national study with a larger sample size in the UK has compared five SARS-CoV-2 immunoassays, four of which have also been validated in the national study in Denmark. However, the UK study reported higher sensitivity and specificity.^142^ The differences may be explained by various factors. First, as for the same type of assays, operation skills, and individual factors of patients, such as different SARS-CoV-2 infection stages, different basic diseases, or immune dysfunction, which might cause the difference in antibody titers, could all exert a certain influence on the accuracy of antibody detection. As for the two above-mentioned national studies, the different validation performances of the same immunoassays may be caused by the difference in the sample. In the UK study, most of the samples were obtained at least 20 days after symptom onset. However, a large proportion of milder cases were included in the Denmark study. Other studies have also worked on the comparison of different approved antibody testing kits, which also affirmed the acceptable performance ability of ELISA/CLIA/ECLIA. In fact, despite the potential above-listed contributing factors, the selection of antigen sites, antibody affinity, immunoassay principles, such as the enzymatic/chemiluminescent/fluorescent labels, signal amplification systems, and others can also result in different sensitivity and specificity among different antibody detection methods. Therefore, there is a need for well-defined international quality standards for the clinical use of SARS-CoV-2 antibody immunoassays.

#### Lateral-flow assay (LFA) used for the rapid SARS-CoV-2 serological detection

Apart from ELISA and CLIA/ECLIA, the lateral-flow assay (LFA) is a paper-based platform for the rapid detection and quantification assay that is wildly used in the antibody and antigen detection of pathogens (like the influenza virus,^143^ HBV,^144^ and HCV^145^). Generally, the platform is composed of three parts: sample pad, conjugate pad, and detection pad. The sample pad makes the sample suitable to bind to the capture components. The conjugate pad contains components that are specific to the sample antibodies and are conjugated to colored or fluorescent particles (like colloidal gold and latex microspheres).^146^ The detection pad commonly includes two lines: a control line and a test line with specific antibodies or antigens immobilized in lines. Color changes of both test and control lines indicate positive results, while negative results are defined as no response of both test and control lines. Given the outbreak’s dynamic, in the context of the lack of portable serologic detection instruments, rapid and accurate testing in public places (such as airports and train stations) remains a challenge. Thus, various types of the LFA assay have been developed for COVID-19 detection to accomplish rapid serological testing, which mostly targets the IgM and/or IgG of SARS-CoV-2 with high sensitivity (Supplementary Table S1). For example, the COVID-19 IgG/IgM Rapid Test Cassette kit from Orient Gene Biotech can detect IgM/IgG with a sensitivity up to 95.8%,^147^ and the Onsite CTK Biotech COVID-19 split IgG/IgM Rapid Test (CTK Biotech, Poway, CA, USA) was able to diagnose COVID-19 with sensitivity and specificity of 88.2% and 94.0%, respectively.^148^ However, in most publications, a satisfying sensitivity of LFA was only occurred after at least one week of the infection due to the low concentration of both IgG and IgM in the first weeks. A special kit targeted the highly conserved nucleoprotein antigen of SARS-CoV-2 with sensitivity and specificity of 57.6% and 99.5%,^149^ respectively. However, according to a meta-analysis evaluating the diagnostic performance of COVID-19 serological assays in early infection, the pooled specificity of LFA was lower than ELISA (*P* = 0.021) but with comparable sensitivity.^150^ Therefore, with the characteristics of the comparable sensitivity to that of ELISA, short turn-around-time, low-cost equipment, and easy-operating, LFA is expected to be a point-of-care tool for the investigation of the serological prevalence of COVID-19.

At present, the serological detection methods based on different principles have the advantages of fast, stable, low-cost, large-scale operation, a high degree of automation, the safety of testing samples, and so on. Although the value of the timely diagnosis of COVID-19 in the early stage of the disease is limited due to the existence of the serum seroconversion time, antibody detection provides the possibility to make up for the risk of missed detection in suspected cases with negative nucleic acid results and evaluate the course of the disease, predicting the prognosis. In addition, antibody dynamic monitoring in asymptomatic infected people who have been in close contact with patients is of great significance for epidemiological investigation. The use of the vaccine has further increased the demand for antibody testing. However, the accuracy and consistency of the detection reagents will affect the results, and some immune cross-reactions will also cause interference. A large number of evaluations and calibration among detection reagents are still needed in the future.

### Antigen testing

Antigen detection means identifying fragments of the SARS-CoV-2 viral surface proteins, which helps with the early diagnosis of the SARS-CoV-2 infection.^151^ The main structural proteins of SARS-CoV-2 include the nucleocapsid protein (N), spike protein (S), envelope protein (E), and membrane protein (M).^152,153^ The detection of SARS-CoV-2 protein in different types of samples is helpful to quickly classify patients with susceptibility to COVID-19 infection and has advantages in shortening the turn-around time and reducing the cost.

Several antigen tests approved for the rapid detection of SARS-CoV-2 have been developed into commercially available tests, primarily performed on the basis of LFA methods.^154^ Despite the simplicity and convenience, the performance capacity of antigen tests widely differs. Four SARS-CoV-2 antigen tests were compared in parallel nasopharyngeal/oropharyngeal swabs from 87 consecutive patients, out of which two tests correctly identified the subjects with high viral loads and three out of four tests detected more than 80% of the subjects with a Ct <30, which is considered as a threshold for infectivity. However, one investigated test had a poor clinical performance.^155^ Bruzzone et al. have quantified the performance of seven different available types of antigen-detecting rapid diagnostic tests compared with RT-qPCR, and the results showed that the overall sensitivity and specificity of antigen tests were 78.7% and 100%, respectively, and a wide range of sensitivity of different brands (66.0–93.8%) was observed.^156^ Therefore, further investigations and confirmatory studies are needed for the validation of different antigen-detection kits.

Antigen detection is usually highly specific, but it is usually not as sensitive as nucleic acid detection.^157^ The sensitivity of the antigen test is higher when the virus loads of nasopharyngeal or oropharyngeal swab specimens are high, mainly during the first week of SARS-CoV-2 infection.^158–160^ Therefore, it cannot be used as the only basis for the diagnosis and exclusion of COVID-19. However, due to its low cost, rapid results, and large-scale deployment, antigen detection can be used in the auxiliary screening of suspected patients, screening of asymptomatic high-risk groups, and regular surveillance, especially in high epidemic situations.

### Nanobiosensor for novel human coronaviruses (HCoVs) detection

**Fig. 3:**
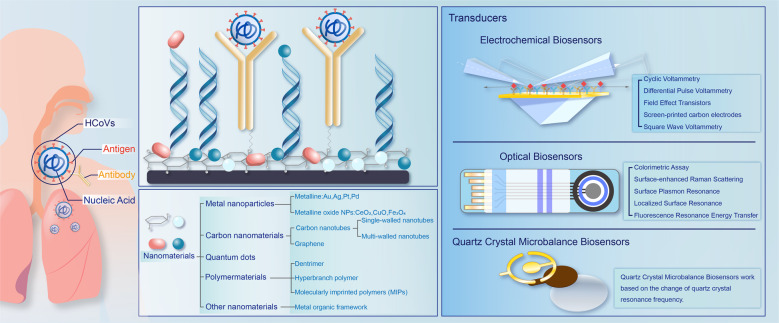
A scheme of several nanobiosensors for the detection of HCoVs. The detection target contains nucleic acids, antigens and antibodies. In biosensors, nanomaterials, such as metal nanoparticles, carbon nanomaterials, quantum dots, polymer materials, and others are utilized. The transducers, including electrochemical biosensors, optical and quartz crystal microbalance sensors, transduced the reaction of receptor and target to electrical, visible, and other measurable signals

Although the methods based on rRT-PCR are currently common techniques for the detection of the novel human coronaviruses (HCoVs) in the clinical laboratory,^14,161,162^ the need for procedures that are highly sensitive^163^ and time-saving pushed the efforts towards the considerable development of precise, efficient, and low-cost devices.^164–166^ Tools like nanotechnology-based biosensors can enhance the performance of SARS-CoV-2 detection.^167,168^ The emerging biosensing-based platforms are promising appliances that are highly specific and sensitive.^169–172^ The biosensors generally combine receptors and transducers,^173,174^ such that a signal change that is generated after the specific interaction between immobilized receptors and targets can be transduced into measurable or visible output.^175,176^ Remarkably, the nanotechnology-based biosensors can achieve higher sensitivity, since the nanomaterials used in the transducers have the advantage of distinctly amplifying the detection signals.^177^ Applied functional nanomaterials binding with receptors are designed and fabricated in a wide range, such as metal nanoparticles (gold nanoparticles, AuNPs),^178–180^ carbon materials (nanotubes,^181^ graphene^182^), quantum dots (QDs),^183^ polymer materials,^184^ and other unique nanomaterials.^185^ Receptors immobilized on the nanomaterials, such as nucleic acids,^186^ antigens,^187^ aptamers,^188,189^ antibodies,^190^ and other biological or synthetic molecules serve as the recognition elements for the targets with certain affinities and specifications.^191^ When it comes to the detection of HCoVs, such as SARS-CoV,^192^ the Middle East respiratory syndrome coronavirus (MERS-CoV)^193^ and SARS-CoV-2^194^ that caused epidemics or pandemics, various transducers based on different principles can be applied in the biological and medical fields, including electrochemical, fluorescence-based or colorimetric biosensors,^195–199^ localized surface plasmon resonance (LSPR),^200–202^ surface-enhanced Raman scattering (SERS),^203–205^ quartz crystal microbalance (QCM) sensors,^206,207^ piezoelectric sensors,^208^ and other kinds of biosensors.^209,210^ Figure 3 shows the schematic diagram of nanobiosensing-based platforms for COVID-19.

#### Electrochemical nanobiosensors used for HCoVs detection

Electrochemical nanobiosensors represent a promising type of nanobiosensors, which operate based on the electrical current passing through electrodes by electrochemical reactions.^211,212^ Due to their advantages, including the high sensitivity, stability, time- and cost-saving,^213^ they have been developed as practical point‐of‐care applications to target the nucleic acids, proteins, cells, and viruses.^214^ Various electrochemical detection methods, such as the field-effect transistor (FET),^215^ differential pulse voltammetry (DPV),^216^ cyclic voltammetry (CV),^217^ and others have been used for the detection of different types of coronaviruses,^218^ as listed in Table 2.

**Table 2:** Several nanotechnology-based biosensors for HCoVs detection

Biosensors			Target		Limit of detection (LoD)	Linear range (LR)
Principle		Nanomaterials	Target virus	Target molecular		
Electrochemical biosensors	FET	In_2_O_3_ nanowire	SARS-CoV	N protein	Sub-nanomolar concentration with 44 µM bovine serum albumin	NA
		Carbon nanotubes	SARS-CoV	N protein	Physiological conditions	NA
		Graphene sheets	SARS-CoV-2	Spike protein	2.42 × 10^2^ copies/mL	NA
	DPV and CV	Fluorine-doped tin oxide (FTO) electrode with AuNPs	SARS-CoV-2	Spike protein	10 fM	1 fM–1 μM
		P-sulfocalix arene (SCX8) functionalized graphene (SCX8-RGO)	SARS-CoV-2	ORF1ab gene	200 copies/mL	NA
Optical biosensors	Lateral flow assay	AuNPs	SARS-CoV-2	IgG and IgM	NA	NA
	Lateral flow assay	AuNPs	SARS-CoV-2	IgG and IgM of N protein	NA	NA
	Lateral flow assay	Selenium-decorated nucleoprotein	SARS-CoV-2	IgG and IgM	5 ng/mL (IgG) 20 ng/mL (IgM)	NA
	Surface-enhanced Raman scattering-based lateral flow immunoassay	Ag shell on SiO_2_ core	SARS-CoV-2	IgG and IgM of the spike protein	1 pg/mL	NA
	Lateral flow assay	Lanthanide-doped polystyrene	SARS-CoV-2	IgG	NA	NA
	PPT, LSPR	AuNIs	SARS-CoV-2	Nucleic acid	0.22 pM	NA
	Colorimetric assay	AuNPs	SARS-CoV-2	Nucleic acid	0.18 ng/L	0.2–3 ng/L
	SPR	AuNPs-based polypeptides	SARS	Coronaviral surface antigen	200 ng/ml	NA
	LSPR coupled fluorescence	AuNPs	SARS-CoV	N protein	0.08 ng/mL	NA
	LSPR	AuNPs	SARS-CoV-2	Spike protein	0.22 pM	NA
	LSPR, Colorimetric assay	AuNPs	MERS-CoV	DNA	1 pmol/L	NA
Piezoelectric Biosensors			SARS-CoV-2	Spike protein	NA	NA
			SARS-CoV-2	Spike protein	NA	NA

FET-based nanobiosensors consist of two parts: the sensing part, which captures the targets with immobilized receptors, and the transducing part, which detects the conductance produced by electrons accumulating on the sensing parts.^219,220^ The spike protein of SARS-CoV-2 has been detected from nasopharyngeal swabs of COVID-19 patients using FET formed by graphene sheets wrapped with the antigen,^221^ with an LoD of 2.42 × 10^2^ copies/mL.^222^ Besides, two biosensors separately equipped with an In_2_O_3_ nanowire^192^ and carbon nanotubes^223^ were designed to detect the N protein of SARS-CoV. The use of these biosensors has greatly reduced the response time, as they respond rapidly within minutes compared with ELISA that takes hours.^192^

The electrochemical biosensing of CV and DPV are widely employed to monitor the electron transfer-initiated chemical reactions in molecular detection.^224^ These nanobiosensors were also applied to detect the SARS-CoV-2 spike protein and nucleic acid. A previous study has reported the use of a nanobiosensor based on these two techniques to detect the spike protein of SARS-CoV-2 with high sensitivity and a linear range from 1 fM to 1 μM.^225^

Following the principle of DPV, a super sandwich-type electrochemical nanobiosensor based on p-sulfocalix arene (SCX8) functionalized graphene (SCX8-RGO) was developed and simultaneously equipped with a smartphone application for the detection of SARS-CoV-2 without RNA amplification. The biosensor manifested an LoD of 200 copies/mL in the clinical settings, and the detection ratios were higher than those of rRT-PCR, suggesting the great potential of this nanobiosensor to be developed as a point-of-care (POC) test in the future.^226^

#### Optical nanobiosensors used for HCoVs detection

Optical nanobiosensors are photonic devices^227^ that are designed based on a wide range of principles, including colorimetry,^228^ light scattering,^229^ fluorescence,^230^ and others. They have various types of output forms, especially for naked-eye detection^231^ (Table 2).

Colorimetric nanobiosensors are auspicious and advantageous optical sensors due to the potentiality of observing by naked eyes.^232,233^ Various LFA strips featured with nanomaterials have been recently developed and utilized in the detection of SARS-CoV-2. With the characteristics of chemical stability, water solubility, and shape controllability, metal nanoparticles have been widely used in gold nanoparticles (AuNPs) as markers immobilized in a conjugate release pad.^234^ LFA strips with these AuNPs were developed to detect IgG, as well as simultaneously detecting both IgG and IgM of SARS-CoV-2 with satisfying diagnostic accuracy.^235,236^ Besides AuNPs, LFA strips based on other nanoparticles, such as selenium-decorated nucleoprotein,^237^ Ag shell on SiO_2_ core,^238^ and lanthanide-doped polystyrene^239^ were designed to detect antibodies (IgG and/or IgM) of SARS-CoV-2, achieving superior speed and sensitivity.

A colorimetric nanobiosensor based on the double-stranded DNA (dsDNA) self-assembly shielded AuNPs was developed to detect MERS-CoV, which targets partial genomic regions (30 bp) of MERS-CoV. It takes no more than 10 min to verify the presence of MERS-CoV without the use of electrophoresis or other operations.^240^ For SARS-CoV-2, a colorimetric biosensor has been proposed based on modifications in the surface plasmon resonance for the naked-eye detection of SARS-CoV-2 within 10 min from the isolated RNA samples utilizing AuNPs functionalized with antisense oligonucleotides (ASOs) that are specific for two regions within the N-gene of SARS-CoV-2. The device can selectively and specifically detect SARS-CoV-2 since no noticeable change in the absorbance was observed with MERS-CoV when it was tested against the MERS-CoV viral RNA load.^241^

Surface plasmon resonance (SPR) nanobiosensors represent a type of optical nanobiosensors, which are based on the principle of the refractive index change near-surface when the biomolecules bind to the reaction surface.^242–244^ As for the antigen detection, SPR nanobiosensors were designed to recognize the surface antigen of SARS using AuNPs-based polypeptides and they achieved a high detection sensitivity with an LoD of 200 ng/mL.^245^

The localized surface plasmon resonance (LSPR) is another optical nanobiosensor, which operates based on the principle of transducing changes in the local refractive index via a wavelength-shift measurement. As a result, it serves as a satisfactory candidate for the real-time detection of biological and chemical analytes.^246,247^ Regarding SARS-CoV-2, an opto-microfluidic sensing platform based on LSPR was developed to identify the antibodies of the SARS-CoV-2 spike protein using AuNPs conjugated with spike proteins. A detection limit of 0.22 pM was accomplished by the sensors using tiny amounts of samples in <30 min.^248^ Furthermore, LSPR-coupled fluorescence with AuNPs as an amplifier has also been introduced to detect the nucleocapsid protein (N protein) of SARS-CoV with a linear range from 0.1 pg/mL to 1 ng/mL.^249^ In addition, a dual-functional nanobiosensor integrated on a chip with two-dimensional nanoabsorbers (AuNIs) combining the plasmonic photothermal (PPT) and LSPR sensing transduction has been developed for the detection of the SARS-CoV-2 viral nucleic acid. This nanobiosensor showed a high sensitivity toward the selected SARS-CoV-2 sequences, including RdRp, ORF1ab, and the E gene sequence, with a low LoD of 0.22 pM, which allows the highly sensitive, precise, and fast detection of specific targets for SARS-CoV-2.^250^

#### Quartz crystal microbalance nanobiosensors used for HCoVs detection

Quartz crystal microbalance (QCM) nanobiosensors with nanocrystals are used to detect molecular targets. They work based on the change of the quartz crystal resonance frequency that is caused by receptors conjugated with the target.^251,252^ These biosensors were successfully applied to detect the antigen of the spike protein of both SARS-CoV^253^ and SARS-CoV-2,^254^ and they achieved a satisfying detection limit (Table 2).

## New biomarkers-based laboratory findings for COVID-19 detection

The development of high-throughput omics detection platforms introduced bioinformatics operations based on molecular maps of genomes, transcriptomics, proteomics, and metabolites, which can provide new opportunities for the screening of novel molecular markers for COVID-19. Table 3 summarizes the COVID-19 studies about multi-platform omics biomarkers.

**Table 3:** COVID-19 studies about multi-platform omics biomarkers

Study description and reference	Population	Sample type	Methods	Comparison to which changes refer	Findings	Significance
Genome-wide Association Study of Severe Covid-19 with Respiratory Failure	Italian; Spanish	Whole blood or buffy coats	Genome-wide association study	Patients with Covid-19 and severe disease	Cross-replicating associations with rs11385942 at locus 3p21.31 and with rs657152 at locus 9q34.2; At locus 3p21.31, the association signal spanned the genes SLC6A20, LZTFL1, CCR9, FYCO1, CXCR6, and XCR1. The association signal at locus 9q34.2 coincided with the ABO blood-group locus.	The authors identified a 3p21.31 gene cluster as a genetic susceptibility locus in patients with Covid-19 with respiratory failure and confirmed a potential involvement of the ABO blood- group system.
Initial whole-genome sequencing and analysis of the host genetic contribution to COVID-19 severity and susceptibility	China	Whole blood	Deep whole-genome sequencing and genetic variants identified	Asymptomatic, mild, moderate, severe, and critically ill patients	Loss of function variants in GOLGA3 and DPP7 for critically ill and asymptomatic disease demonstration has a potential monogenic effect; the most significant gene loci associated with severity were located in TMEM189 UBE2V1 that are involved in the IL-1 signaling pathway. The p.Val197Met missense variant that affects the stability of the TMPRSS2 protein displays a decreasing allele frequency among the severe patients; the HLAA*11:01, B*51:01, and C*14:02 alleles significantly predispose the worst outcome of the patients.	The authors highlighted genes and variants that may help guide targeted efforts in containing the outbreak.
The noncoding and coding transcriptional landscape of the peripheral immune response in patients with COVID-19	China	Whole blood	Multi-transcriptome sequencing	Moderate and severe COVID-19 patients	MiR-146a-5p, miR-21-5p, miR-142-3p, and miR-15b-5p as potential contributors to the disease pathogenesis, possibly serving as biomarkers of severe COVID-19 and as candidate therapeutic targets; the transcriptome profiles consistently suggested hyperactivation of the immune response, loss of T-cell function, and immune dysregulation in severe patients.	This research provided a comprehensive view of the noncoding and coding transcriptional landscape of peripheral immune cells during COVID-19, furthering our understanding and offering novel insights into COVID-19 pathogenesis.
Ultra-high-throughput clinical proteomics reveals classifiers of COVID-19 infection	Germany	Plasma and serum	Ultra-high-throughput proteomics on ISO13485 platform and high-flow liquid chromatography	COVID-19 patients and healthy volunteers	Alpha-1B-glycoprotein (A1BG), beta and gamma-1 actin (ACTB; ACTG1), monocyte differentiation antigen and lipopolysaccharide co-receptor CD14, lipopolysaccharide-binding protein (LBP), galectin 3 binding protein (LGALS3BP), leucine-rich alpha-2-glycoprotein (LRG1), haptoglobin (HP), protein Z-dependent protease inhibitor (SERPINA10), apolipoprotein C1 (APOC1), gelsolin (GSN) and transferrin (TF) as potential COVID-19 severity biomarkers.	The authors highlighted the role of complement factors, the coagulation system, several inflammation modulators as well as pro-inflammatory signaling both upstream and downstream of interleukin 6 and identify prognostic biomarkers for COVID-19. The work also provided evidence that proteomic signatures have the potential to outperform conventional clinical assays.
Serum proteomics in COVID-19 patients: altered coagulation and complement status as a function of IL-6 level	America	Serum	Nano ultra-high-pressure liquid chromatography-tandem mass spectrometry metabolomics	Positive and negative COVID-19 patients and convalescent patients	Significant dysregulation in serum levels of various coagulation factors accompanied by increased levels of anti-fibrinolytic components, including several serine protease inhibitors (SERPINs).	The authors highlighted a clear increase in the levels of inhibitory components of the fibrinolytic cascade in severe COVID-19 disease, providing potential clues related to the etiology of coagulopathic complications in COVID-19 and paving the way for potential therapeutic interventions, such as the use of pro-fibrinolytic agents.
Plasma Proteomics Identify Biomarkers and Pathogenesis of COVID-19	China	Plasma	LC-MS/MS	Patients with fatal outcome, patients diagnosed with severe symptoms, and patients diagnosed with mild symptoms	A compact biomarker combination containing 4 proteins, including orosomucoid-1/alpha-1-acid glycoprotein-1 (ORM1/AGP1), ORM2, fetuin-B (FETUB), and cholesteryl ester transfer protein (CETP) for the classification of COVID-19 patients and H volunteers; a biomarker combination containing the 3 proteins of CETP, S100A9 and C-reactive protein (CRP) to predict different clinical outcomes.	This research provided valuable knowledge about COVID-19 biomarkers and shed light on the pathogenesis and potential therapeutic targets of COVID-19.
Serum Protein Profiling Reveals a Landscape of Inflammation and Immune Signaling in Early-stage COVID-19 Infection	China	Plasma	High-density antibody microarray	Early COVID-19 and influenza patients	A total of 125 differentially expressed proteins associated with viral infection, inflammation, immune cell activation, and migration, in addition to the complement and coagulation processes; changes in the complement and coagulation cascades in early infection and severe COVID-19 patients; activation of viral infection pathways (MAPK, ERK1/ERK2, JAK-STAT, PI3K) in acute infection.	This research is valuable to understand the COVID-19 pathogenesis, identification of biomarkers, and development of the optimal anti-inflammation therapy.
Transcriptional and proteomic insights into the host response in fatal COVID-19 cases	China	Fixed paraffin- Embedded Lung and Colon Samples	Total RNA sequencing (RNA-Seq)	Patients who died of COVID-19	Protease cathepsins B and L and the inflammatory response modulator S100A8/A9 are highly expressed in fatal cases.	This research shed light on COVID-19 pathophysiology and offered potential therapeutic targets for severe COVID-19 disease.
Proteomic and Metabolomic Characterization of COVID-19 Patient Sera	China	Serum	LC-MS/MS; (RP/UPLC)-MS/MS methods with positive ion-mode ESI; RP/UPLC-MS/MS with negative-ion-mode ESI; HILIC)/UPLC-MS/MS with negative-ion-mode ESI	Severe and non-severe patients; non-COVID-19 patients with similar clinical characteristics and negative nucleic acid test	A total of 22 proteins and 7 metabolites were identified to establish a model for distinguishing severe infections; molecular changes implicating dysregulation of macrophages, platelet degranulation, complement system pathways, and massive metabolic suppression.	This research offered a landscape view of blood molecular changes induced by the SARS-CoV-2 infection, which may provide useful diagnostic and therapeutic clues in the ongoing battle against the COVID-19 pandemic.
Multi-Omics Resolves a Sharp Disease-State Shift between Mild and Moderate COVID-19	Hispanic or Latino; White, Asian; Black or African American; Native Hawaiian or other Pacific Islander; American Indian or Alaska Native; no race recorded	Plasma	ProSeek panels (Olink Biosciences, Uppsala, Sweden); Metabolon’s ultra-high-performance liquid chromatography/tandem mass spectrometry (UHPLC/MS/MS) Global Platform; Chromium Single Cell Kits (10x Genomics)	COVID-19 patients; healthy controls	A major immunological shift between mild and moderate infection, which includes an increase in inflammation, drop in blood nutrients, and the emergence of novel immune cell subpopulations that intensify with disease severity.	This study suggested that moderate disease may provide the most effective setting for therapeutic intervention.
Blood molecular markers associated with COVID-19 immunopathology and multiorgan damage	China	Peripheral blood and plasma samples	LC-MS/MS; NMR spectroscopy	COVID-19 patients; healthy controls.	The tricarboxylic acid cycle (TCA) and glycolytic pathways were significantly downregulated in both mild and severe patients. In patients with COVID-19, the expression of host defense pathways was increased, such as T-cell receptor signaling pathways.	This study suggested that gene proteins and exRNAs are potential biomarkers that may be helpful in predicting the prognosis of SARS-CoV-2 infection.
Omics-Driven Systems Interrogation of Metabolic Dysregulation in COVID-19 Pathogenesis	China	Plasma	A combination of targeted and untargeted tandem mass spectrometry	Mild, moderate, and severe COVID-19 patients and healthy controls	Enhanced levels of sphingomyelins (SMs) and GM3s; reduced diacylglycerols (DAGs); COVID-19 patients with elevating disease severity were increasingly enriched in GM3s.	This study suggested that GM3-enriched exosomes may partake in pathological processes related to COVID-19 pathogenesis and presented the largest repository on the plasma lipidome and metabolome distinct to COVID-19.

*LC-MS/MS* liquid chromatography-tandem mass spectrometry, *RP/UPLC* reverse-phase/ultra-performance liquid chromatography, *ESI* electrospray ionization, *HILIC* hydrophilic interaction liquid chromatography.

### Genomics and transcriptomics molecular markers

The host’s genetic background is associated with the immune response, severity, and susceptibility. Understanding the genetic background will help us to make early predictions and choose the best treatment options for the clinical trials. Transcriptomics, which are the downstream pathways of the genome, can reflect the changes in the transcripts in cells or tissues and are more directly related to the pathophysiological processes of the diseases.

Studies in Spain and Italy have found loci that are significantly associated with severe disease on chromosomes 3 and 9.^255^ Based on the in-depth sequencing and analysis of Chinese patients and normal control patients, the specific haplotype of the human leukocyte antigen region (HLA) of chromosome 6 and the function loss of GOLGA3, DPP7, and other genes were found to increase the risk of developing severe COVID-19.^256^ However, many other gene polymorphisms that are significantly associated with severe illness cannot be replicated in populations with different genetic backgrounds, which may have different post-infection symptoms and severity; thus, further research and comparison are still needed.^256,257^

The analysis of transcripts from various model systems (in vitro tissue culture, in vitro primary cell infection, and peripheral blood mononuclear cells from COVID-19 patients) revealed that compared with other common respiratory viruses, the overall transcription imprinting of the host of SARS-CoV-2 infection is abnormal, and the interferon and inflammatory response-related molecules are associated with the SARS-CoV-2 infection of the main sign.^258–260^ In addition to the encoding part of the human genome, noncoding RNA (ncRNA) also shows great potential to play a role in various cellular processes. Radhakrishnan identified several differentially expressed MALAT1, along with the long ncRNA (lncRNA) NEAT1 during the course of infection, which could serve as disease biomarkers.^261^ The work of Tang et al. revealed that mir-15B-5P was a specific gene for severe COVID-19 infection and could be used as a potential biomarker through the comprehensive analysis of noncoding and coding transcription profiles.^262^ The micro RNAs of mir-146a-5p, mir-21-5p, and nir-142-3p are potential biomarkers for the severity of COVID-19 and may be involved in the overactivation of immune and inflammatory responses, loss of T-cell function, and immune regulatory disorders in patients with severe COVID-19 infections. Although the transcriptome research shows more COVID-19 immune molecules and cells in the process of clinical features, the results involving more related molecular mechanisms can be more systematic if the clinical phenotype can learn and integrate the transcriptome data analysis, resulting in more comprehensive conclusions.

### Molecular markers of proteomics, metabolomics, and lipidomics

Proteins are substantial bases to maintain vital movement; they possess an important biological function and can directly reflect gene expression. Protein biomarkers of infectious diseases have been traditionally defined based on immunological categories mostly.^263^ However, the large-scale, high-throughput, and high-sensitive detection of proteomic signatures of multiple biological samples, such as urine, plasma, and serum, can provide a broader host-response profile to efficiently screen for disease biomarkers. A number of studies on proteomics have recently shown the characteristic changes of related potential blood biomarkers, such as the complement factors, coagulation factors, and inflammatory regulators, in severe COVID-19 cases.^264–269^ Moreover, the severity of COVID-19 can be assessed by constructing a clinical classifier using the proteomic signature, which has been proven to have an accurate predictive efficiency.^235^

The metabolome represents the downstream event of the genome and proteome. It can reflect the changes in the cellular function of biological systems before and after virus infection, while the lipids participate in multiple steps of the virus life cycle and play many indispensable roles in the cell functions. Therefore, exploring the composition and content of lipids and small molecule metabolites in COVID-19 patients represents another effective tool to find molecular markers. In the work of Wu et al. and Shen et al., dyslipidemia was observed in COVID-19 patients, which was consistent with the severity of the disease, but some variation trends (PC) were different between the two studies.^235,270^ Metabonomics studies have shown profound changes in the malic acid of the trichloroacetic acid cycle, carbamyl phosphate of the urea cycle, and guanosine of the nucleotide biosynthesis in COVID-19 death.^270^ In addition, a variety of metabolites that are associated with multiple viral infections and pathogenesis have also been determined. However, their repeatability and consistency still need to be confirmed by large-scale comprehensive studies.

### Human microbiome as a potential marker

The infection of COVID-19 is characterized by progressive inflammation, which then develops multiorgan dysfunction.^271–273^ Therefore, the progression of COVID-19 is relevant to both virus invasion and host immune response.^271^ Various factors affect the immune response to SARS-CoV-2, but several researchers interestingly investigated how the microbiome affected the immune response.^274^ The human microbiome represents the microorganisms that colonized the human body,^275^ which participate in human activities.^276–280^ Dysbiosis microbiome may create an inflammatory response caused by SARS-CoV-2 and may even trigger a cytokine storm, which is a progressive multiorgan inflammatory damage.^281^ Moreover, the microbiome was recognized as a potential marker for COVID-19 susceptibility, severity, and prognosis.^282^

An increasing number of studies have focused on the gut microbiome, and the potential effect mechanism of the gut microbiome in the COVID-19 infection is beginning to be elucidated.^132,283^ Angiotensin-converting enzyme 2 (ACE2) is a reported cellular receptor of SRAS-CoV-2. During viral infection, the spike glycoprotein is capable of binding to ACE2.^284,285^ ACE2 is a type ACE2, which is a type I membrane protein expressed in lungs, heart, kidneys, intestine, and even on the ocular surface.^286,287^ ACE2 coupled with B^0^AT1 plays a role in the transport of neutral amino acids, such as tryptophan.^285,288^ Tryptophan can affect the expression of antimicrobial peptides that influence the gut microbiome.^289,290^ Several studies have found a reduction of ACE2 expression in the gastrointestinal tract in SARS-CoV.^291,292^ As a consequence, the decrease in ACE2 may account for the decrease in tryptophan absorption in the gastrointestinal tract. This may account for the gastrointestinal symptoms caused by SARS-CoV-2, such as diarrhea, and may be a potential regulatory mechanism of the gut microbiome.^132,293^ The alteration of the gut microbiome existed persistently even after recovery from COVID-19.^294^ A prospective study conducted by Chen et al. reported unrestored microbiome richness after a 6-month recovery in the gastrointestinal tract.^295^ This may illustrate that a subset of recovered COVID-19 patients complained of persistent fatigue, dyspnea, and joint pain.^296^ The gut microbiome has potential protective effects in the COVID-19 infection due to the decrease in several bacteria that are correlated with increasing cytokines and chemokines (such as TNF-α, CXCL10, CCL2, and IL-10) involved in overaggressive inflammation.^296^

The dysbiosis in gut microbiome mainly exists when the diversity of microorganism is decreased,^297^ but the dysbiosis of human-associated microbiota can also show as a change in the microbial composition. Enaud et al. described that the gut and lungs have potential communications involving the microbiome via the gut-lung axis.^298–300^ Thus, apart from the role of the gut microbiome in COVID-19, evidence also indicated that changes in lung microbe, especially those enriching gut microbiomes, may predict ARDS (acute respiratory distress syndrome),^301,302^ which is a severe complication of COVID-19. In addition to the gut and lung microbiome, the involvement of oral microbiomes during the SARS-CoV-2 infection was also discussed. Ren et al. compared patients with healthy individuals and detected an alteration of the oral microbiome in confirmed COVID-19 patients. Moreover, a diagnostic model based on 16 oral microbial markers was constructed for COVID-19 diagnosis with a great efficacy (AUC: 98.06%, 95% CI: 96.31–99.82%, *P* < 0.0001).^282^ The oral microbe has also drawn the attention of researchers,^303–305^ as the number of some oral microorganisms persistently increased. Besides, the restoration of the oral microbe was not detected between healthy and recovered individuals, which indicated that the oral microbiome may be involved in the recovery of patients with COVID-19.^282^ Although a lot of attention has been paid to the human microbiome, more investigation is still needed of the specific role of the human microbiome and potential mechanisms in the inflammation process of COVID-19.

## Non-pathogen-based laboratory findings for COVID-19 management: prevention, diagnosis, and treatment

**Fig. 4:**
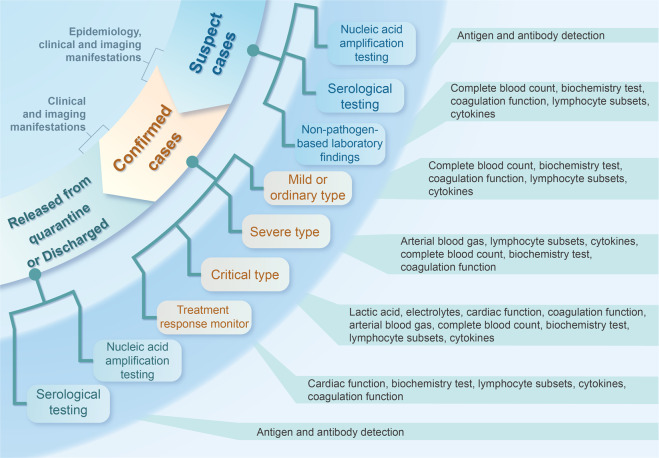
Laboratory inspection selection of COVID-19

Although the direct evidence to reflect SARS-CoV-2 infection is the etiological evidence, the existence of false-negative and false positive results may lead to inappropriate management.^306,307^ Therefore, it is important to use non-pathogen-based laboratory findings in the screening, diagnosis, and differential diagnosis of COVID-19. Such findings can help to predict the disease progression and guide treatment decisions, especially in case the etiological evidence is negative (Fig. 4).^308,309^

### Screening and diagnosis

The early stages of the COVID-19 disease have a broad clinical spectrum and tend to be mild.^310,311^ Detecting the disease in the early stages and interrupting its transmission through extensive screening, rapid identification, and isolation of all infected individuals represent key steps to contain the epidemic.^312,313^ The current rapid global spread of COVID-19 poses a challenge to medical resource allocation, and laboratory indicators-based identification methods will facilitate the aggressive screening, early diagnosis, and effective prevention to minimize the risk of transmission, particularly regarding the isolation and management of asymptomatic cases, of which a high incidence up to 24.2–75% has been reported.^314–318^

As an effective complement to nucleic acid tests, inflammatory indicators can be used for the preliminary differential diagnosis. Many research studies have proved that most patients, including asymptomatic ones, have normal or decreased WBC count, decreased lymphocyte count, as well as standard platelet count and hemoglobin level at the early stage of the disease.^319–322^ Tan and colleagues showed the eosinophil counts in COVID-19 patients to be significantly lower than those in patients with respiratory tract infections who had negative viral nucleic acid tests at fever clinics during the same period, which can help to rapidly detect and identify patients with similar manifestations of respiratory infections.^323,324^ In addition, most patients had elevated levels of the C-reactive protein (CRP), erythrocyte sedimentation rate (ESR), and ferritin, such that CRP increases progressively with the disease exacerbation.^325–327^ Procalcitonin (PCT) can be used to identify bacterial pneumonia.^328–330^ These laboratory findings could also be used to construct prediction models that can translate research findings into valuable tools to be used in supporting prompting screening programs and clinical decision-making on the diagnostic pathway.

### Predicting disease progression

Laboratory predictors that can help with the early and accurate identification of patients at risk of progressing to severe disease can improve the patient outcomes and help to allocate medical resources rationally. Many studies have suggested that the immune response monitoring for COVID-19 patients, including the detection of cytokines, chemokines, and lymphocyte subsets can be one of the bases to predict the severe transitions of patients.^331–333^ Besides, IL-2, IL-7, IL-10, and TNF were significantly higher in severe patients compared with non-severe patients.^334^ Abnormal outcomes of lymphocytopenia and leukopenia were more pronounced in severe patients compared with non-severe patients.^335^ As the disease progresses, the CD4 + , CD8 + , and CD3 + T-cell subsets continued to decrease, and the ratio of neutrophils count/CD8 + T-cell count and that of neutrophils count/lymphocyte count can be used to predict the severity of the COVID-19 infection.^316,336–338^ In addition, a few studies have shown that the serum amyloid A (SAA) levels in patients with severe or mild diseases are statistically significant (*P* = 0.003), which indicates that SAA has a certain predictive value. Nevertheless, it still needs to be confirmed using larger sample size and further studies.^339–341^

### Guiding treatment decisions and monitoring the response to treatment

A comprehensive interpretation of the laboratory findings can also help in the treatment decision-making, monitoring the response to treatment, and identifying early possible complications. Recent studies have shown that the relative indexes of lymphocyte proportion are important to the prognosis and therapeutic reaction.^324,342–344^ The dynamic monitoring of the T-cell percentage and absolute count can help to enhance the clinical understanding of the cellular immune function, thus guiding treatment directions and monitoring immune responses. Antagonizing certain key inflammatory cytokines may also be used as adjuvant therapy. Furthermore, acute respiratory failure syndrome can develop in many severe COVID-19 patients, which can be combined with acute liver, kidney, or cardiac injury, neurological manifestations, and other manifestations of multiorgan failure.^316,345–347^ Therefore, monitoring the organism damage can be greatly enhanced by including the cardiac enzymes, hepatorenal function indicators, blood gas analysis, and other biochemical indicators to make suitable individualized treatment options. It should also be taken into consideration that some therapeutic drugs, such as antiviral drugs and antipyretic and analgesic drugs, have certain hepatorenal toxicity.^348^

These results suggest that laboratory findings could reflect the immune status, disease progression, organism damage, and treatment response, and their reasonable use can provide a more comprehensive evidence for the early screening and diagnosis to predict the disease progression and make individualized treatment options. However, it is important to note that most of the limited published research data are the detection results of unrepeated blood samples after admission or at the early stage of disease and on a small sample; thus, a further in-depth study is still needed.

### Intelligent prediction model-aided system

The large number of variables associated with SARS-CoV-2 infection and disease progression will bring challenges to clinical decision-making. However, the development of data mining and machine-learning techniques has solved this problem to some extent. If we can mine out valuable and universal rules from the accumulated historical data from the early stage and establish an intelligent prediction model that includes representative features, such models will exploit the available information and use fine differences that clinicians cannot recognize from the laboratory findings to provide a powerful tool to assist individualized diagnosis, progression prediction, and treatment options.^349^

**Fig. 5:**
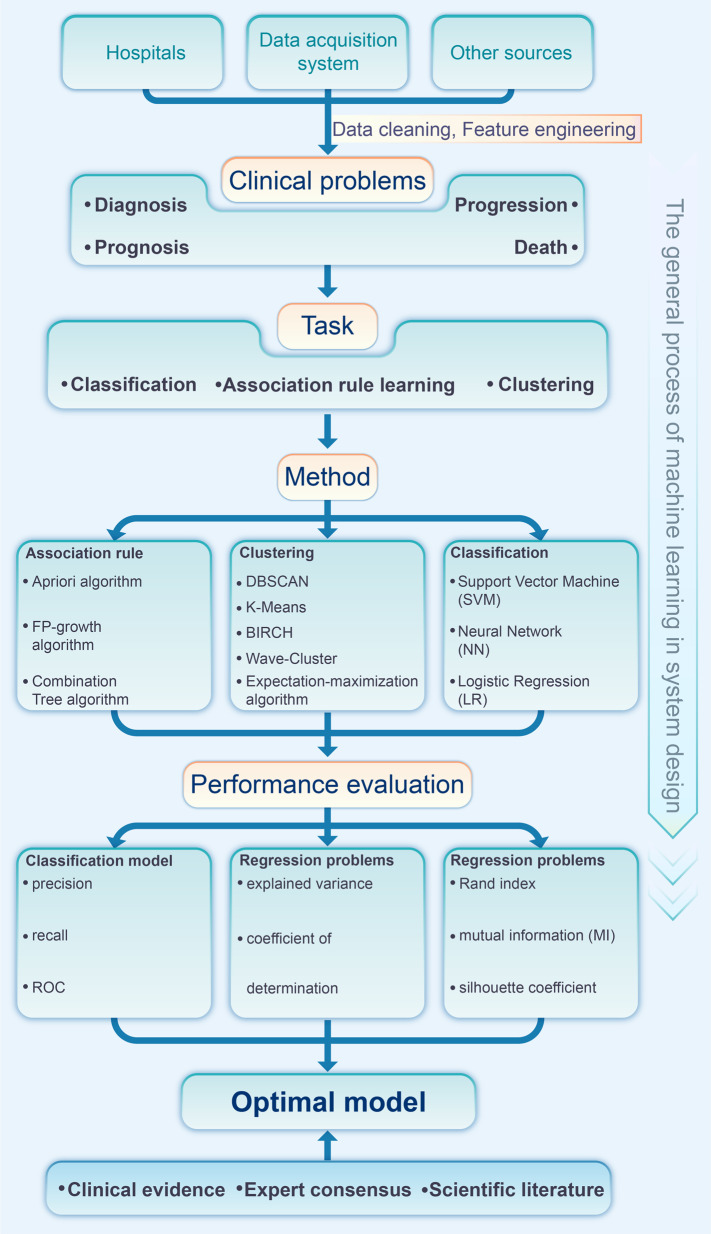
Flow chart of the system design

Although there are still no unified standards to guide the construction of candidate auxiliary intelligent prediction model-aided system for COVID-19, the realization of the clinical application is generally divided into two parts: system design and system implementation.^350–353^ In the system design, there are five core steps (Fig. 5). The patients’ data are collected from different sources and preprocessed. The adequacy of the raw data should be ensured to obtain a satisfactory performance for data mining. Data sampling and cleaning can improve the correctness and efficiency of the model.^354,355^ Then, it is necessary to determine the target problem according to the clinical needs and select the appropriate learning algorithms for the task. Commonly used data mining methods include classification, clustering, and association rule learning.^356–358^ These methods can help to discover similar types of groups or group patterns, extract significant patterns and visualize them.^358^ In this process, feature selection, and the preliminary prediction model construction are completed. Finally, we need to evaluate the properties of the model to decide on the one with the optimal comprehensive performance and verify its effectiveness.

**Fig. 6:**
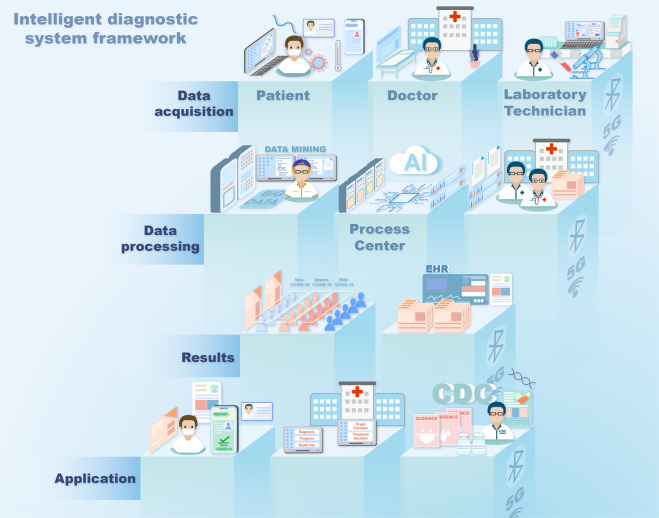
Intelligently assisted individualized diagnosis and treatment system for COVID-19

In the system implementation, the clinical application of the model is realized. Currently, web and smartphone applications are the most effective means of presentation. After registration and login, the users input or transmit the needed data. The system will then produce individualized predictions according to the comprehensive information, thus automatically generating a confirmed, suspected, or suspicious diagnosis, along with the classification into mild, ordinary, severe, or critical infection. At the same time, the system will automatically establish an online real-time updated COVID-19 database. By uploading the data and performing updates and intelligent maintenance, the latest data are used to optimize the intelligent diagnosis model in real time, which improves the accuracy of diagnosis and achieves the purpose of accurate, punctual, shared, and individualized diagnosis and treatment of COVID-19 (Fig. 6).

Several studies have been conducted to detect the SARS-CoV-2 infection, predict the progression of the severe disease and risk of death, and monitor the treatment response.^359–364^ The most common predictors are the above-mentioned relevant hematological indicators, and the number of included variables varies between 3 and 17, such that most of them are concentrated within 7–9 variables.^361,365–372^ The achieved accuracy is generally between 0.69 and 0.99.^361,370,373–378^ In China, the experts proposed a “COVID-19 intelligent diagnosis and treatment program” (nCapp) based on the medical technology of the Internet of Things (IoT) for the early identification, report, isolation, and treatment of COVID-19 patients.^379^ Through “Comprehensive perception → reliable transmission → intelligent processing”, this model represents a decision-oriented big data analysis model that is supported by information technologies, such as communications, electronics, biology, and medicine. It implements real-time and all-round intelligent public health management. It is suitable for hospitals and public health centers of different scales that need to detect, isolate, and manage suspicious patients in a timely manner and cut off all transmission routes, so as to strengthen the prevention and control of the epidemic.

The intelligent prediction model based on laboratory index has been shown to possess many advantages. First of all, the data can be retrieved from the patient’s electronic health record (EHR) without the additional need for material expenses. Second, the practical collection of the information allows a rapid analysis of a large number of patients. Finally, the model can be integrated into the clinical workflow through visualization, such that the analysis results can be directly obtained, which helps to achieve the best efficacy-economic ratio and the most satisfactory medical services in the shortest time and through the least number of intermediate links.

However, we still need to pay attention to the following points. First of all, sufficient sample size and standardized methods should be used for the modeling, and real external verification should be carried out to avoid obvious deviations. Second, a trade-off should be made between the most effective number of predictive variables and achieving a good predictive ability to avoid overfitting. Similarly, a balance needs to be established between the interpretability and accuracy of the model. Although the “black box model” may be more accurate, it poses a higher risk for decision-making, and the clinical environment may favor a more interpretable model. Finally, several studies manage to complete the system design and preliminary model only, while many steps are still needed to obtain the real clinical application. The intelligent prediction model auxiliary system requires close cooperation between the users and managers. Such a reliable system for the prevention, clinical diagnosis, and treatment can only be established by ensuring authentic and reliable data and through continuous training and correction. From a technical point of view, we believe that a good prediction model can be used as a supplement rather than an alternative to molecular detection to assist in the diagnosis, progression prediction and individualized treatment options of COVID-19. The use of such model can reduce the pressure of the clinical parameter monitoring and other related medical burdens. However, clinical decisions cannot be achieved only based on mathematical models, and these models can support the human experts to better serve the patients.

## Conclusions and outlook

In the time of the current pandemic and future epidemics, laboratory testing remains the cornerstone of public health control and mitigation strategies. Continued improvement in the detection methods will provide guidance for the prevention, treatment, and vaccine development. At present, pathogen-based laboratory findings are still the most commonly used direct evidence to judge whether the patients have SARS-CoV-2 infection or not. Nucleic acid test results have a high specificity, but there is the problem of missed detection, especially in the throat swab test of mild infection, as it can easily appear as false negative. More work is needed to achieve simple and efficient release and enrichment of RNA from the clinical samples for direct amplification. Antibody detection, which is easier to manage, can be used as an indirect evidence to judge SARS-CoV-2 infection, evaluate the efficacy of the vaccine, and reflect the current infection status of the patients. At the same time, the progress of the disease course can be comprehensively evaluated according to the type and titer of antibodies. In different stages of infection, nucleic acid and antibody detection had different sensitivity values, especially in the middle and later stages of infection, as the detection rate of nucleic acid decreased, and the detection rate of antibody increased. The combined detection of nucleic acid and antibody can reduce the rate of missed diagnosis. When using antibody detection alone, the interpretation of the results should be cautious, specifically, we need to check the epidemiological history of the patients, whether they have been vaccinated with a SARS-CoV-2 vaccine and whether immune-related underlying diseases exist as complications. The types of samples suitable for antigen detection are generally infected site samples, mainly nasopharyngeal swabs and bronchoalveolar lavage fluid. The detection results are greatly affected by the quality of the sample, site of infection and amount of virus expression. The sensitivity is low, and it is easy to produce false-negative results. At present, it is still necessary to further screen for and prepare antibodies with high affinity and specificity for the development of antigen-detection reagents.

In general, detection methods targeting nucleic acids, antigens, or antibodies will always play an important role. We recommend that future research efforts focus on enhancing the testing capabilities, simplifying the testing process and providing faster results in an easy-to-use format. At present, multiple testing options with potential applications are described in preprints and published articles, including optical and electrochemical nanobiosensors microfluidic chip and so on. The development of point-of-care testing (POCT) has also greatly expanded the application scenarios of the SARS-CoV-2 test. It does not only serve a large number of people living all over the world, especially those in developing countries who lack modern diagnostic facilities but it can also be used to prevent the spread of diseases in a family-based rapid detection effort, which is also applicable in developed countries. It should be recognized that under certain conditions, these methods have some significant advantages.

In addition, the molecular maps of genomes, transcriptomics, proteomics, and metabolites can provide new opportunities for the screening of novel molecular markers for COVID-19, thereby alleviating the current demand for testing methods. Such markers can be used for the early diagnosis and also have a good application prospect in monitoring the course of infection, predicting the disease course, and evaluating the prognosis. An in-depth analysis of the upregulation and downregulation of the expression levels of these markers and the mechanism of action can not only reveal and explain the mechanism of viral infection to the host but also provide a basis for the screening of possible drug targets. Therefore, rapid deployment of clinical decisions based on biomarker data will be a key part of the future development in the fight against the disease. Integrating multiple markers and constructing simple and easy-to-operate decision-making rules have the potential to produce a rapid, economical and efficient screening tools, which are likely to be highly valuable as routine first-line detection. It is worth noting that future research requires sufficient samples to power the research and advanced health informatics methods to turn the data into clinically useful conclusions and observe the performance in a wider range of patients.

With the deployment of clinical research into clinical practice, we have gradually realized that relying on a single test result and blindly emphasizing the high specificity and high sensitivity of the detection methods cannot meet the needs of precise diagnosis, especially when dealing with a complex disease such as COVID-19. The clinical diagnosis and treatment model established based on individualized factors, pathogen-based, and non-pathogen-based laboratory big data is the new direction in precision medicine. On the one hand, close observation of the dynamic changes of integrative results may indicate infection, inflammation, or tissue damage and thus support the diagnosis and have a guiding significance for predicting the outcome of the disease. On the other hand, it can standardize the interpretation of test results, empower grassroots doctors and hospitals, and truly realize hierarchical diagnosis and treatment. However, it is worth mentioning that there is considerable heterogeneity in the accuracy of many biomarkers, and the used cut-off values and reference criteria are poorly described in many cases. In some cases where resources are very limited, some careful choices may be made when making a diagnosis. Future research should focus more on verifying, comparing, improving, and updating promising forecasting models, rather than the development of new models.

Finally, the COVID-19 pandemic has pushed laboratory testing into a new situation, with the addition of big data, artificial intelligence, and other technologies, which are actively promoting the realization of personalized precision medicine. The biosensor detection platform linked to the mobile user terminal system is ideally more convenient to carry and easy to deploy on a large scale, which can play a greater role in the precise diagnosis and treatment. At the same time, accessing the detection information of the digital terminal is also more convenient for the artificial intelligence-based diagnosis system to analyze and evaluate the updated data and provide enhanced diagnosis and treatment decision-making suggestions. These can help to realize a rapid, sensitive, specific and cost-effective diagnosis of COVID-19 by minimally trained individuals and with limited technical infrastructure in developing and developed countries alike. Global solidarity is needed to strongly intertwine infection control and test interventions. The most effective way to meet the current needs for accurate diagnosis and treatment of COVID-19 may be the combination of the promotion and application of testing methods and AI information processing, and the continuous emergence of more creative and multi-faceted detection methods will provide seeds for solutions.

It is important to note that the data on COVID-19 are rapidly evolving as more studies become available, and some of the details in this review may change as many prominent studies have also found weaknesses in their experimental studies and design.

## Supplementary information

Table S1
