# Supplementary material for: Applications of laboratory findings in the prevention, diagnosis, treatment, and monitoring of COVID-19
Source: Signal Transduct Target Ther. 2021 Aug 25;6:316. doi: 10.1038/s41392-021-00731-z (PMC8386162; doi:10.1038/s41392-021-00731-z)
Supplement: Supplementary file 1 — Table S1 [file 41392_2021_731_MOESM1_ESM.docx]

| **Table S1. Several antibody tests with Emergency Use Authorization (EUA)** | | | | | | | |
| --- | --- | --- | --- | --- | --- | --- | --- |
| **Company** | **Kit** | **Antibody** | **Test Type** | **Antigen** | **Sensitivity** | **Specificity** | **Relevent document** |
| Abbott Laboratories Inc. | Architect AdviseDx SARS-CoV-2 IgG II | IgG | CMIA | N | 97.56% | 99.55% | <https://www.fda.gov/media/146371/download> |
| Abbott Laboratories Inc. | Alinity AdviseDx SARS-CoV-2 IgG II | IgG | CMIA | N | 97.50% | 99.60% | <https://www.fda.gov/media/146372/download> |
| Abbott Laboratories Inc. | Alinity i SARS-CoV-2 IgG | IgG | CMIA | N | 100% | 99% | <https://www.fda.gov/media/137910/download> |
| Abbott Laboratories Inc. | Architect SARS-CoV-2 IgG | IgG | CMIA | N | 100% | 99.60% | <https://www.fda.gov/media/137383/download> |
| Abbott Laboratories Inc. | Architect AdviseDx SARS-CoV-2 IgM | IgM | CMIA | N | 95.00% | 99.56% | <https://www.fda.gov/media/142940/download> |
| Abbott Laboratories Inc. | Alinity AdviseDx SARS-CoV-2 IgM | IgM | CMIA | N | 95.00% | 99.56% | <https://www.fda.gov/media/142941/download> |
| Access Bio, Inc. | CareStart COVID-19 IgM/IgG | IgM and IgG | LFA | N and S1 RBD | 98.44% | 98.90% | <https://www.fda.gov/media/140447/download> |
| ACON Laboratories, Inc. | ACON SARS-CoV-2 IgG/IgM Rapid Test | IgM and IgG | LFA | — | IgG 100%  IgM:96.7% | IgG 97.5%  IgM 98.8% | <https://www.fda.gov/media/144562/download> |
| ADVAITE, Inc. | RapCov Rapid COVID-19 Test | IgG | LFA | N | 93.30% | 100% | <https://www.fda.gov/media/145080/download> |
| Assure Tech. (Hangzhou Co., Ltd) | Assure COVID-19 IgG/IgM Rapid Test Device | IgM and IgG | LFA | N and S1 | IgG 90% IgM 100% | IgG 100% IgM 98.8% | <https://www.fda.gov/media/139792/download> |
| Babson Diagnostics, Inc. | Babson Diagnostics aC19G1 | IgG | CLIA | — | 100% | 100% | <https://www.fda.gov/media/139446/download> |
| Beckman Coulter, Inc. | Access SARS-CoV-2 IgG II | IgG | CLIA | S1 RBD | 98.90% | 100.00% | <https://www.fda.gov/media/146901/download> |
| Beckman Coulter, Inc. | Access SARS-CoV-2 IgM | IgM | CLIA | S1 RBD | 98.30% | 99.90% | <https://www.fda.gov/media/142911/download> |
| Beckman Coulter, Inc. | Access SARS-CoV-2 IgG | IgG | CLIA | S1 RBD | 96.80% | 99.60% | <https://www.fda.gov/media/139627/download> |
| Beijing Wantai Biological Pharmacy Enterprise Co., Ltd. | WANTAI SARS-CoV-2 Ab Rapid Test | Total Antibody | LFA | RBD | 100% | 98.8% | <https://www.fda.gov/media/140030/download> |
| Beijing Wantai Biological Pharmacy Enterprise Co., Ltd. | WANTAI SARS-CoV-2 Ab ELISA | Total Antibody | ELISA | RBD | 96.7% | 97.5% | <https://www.fda.gov/media/140929/download> |
| Biocan Diagnostics Inc. | Tell Me Fast Novel Coronavirus (COVID-19) IgG/IgM Antibody Test - Letter of Authorization | IgM and IgG | LFA | N and S | IgG 93.3% IgM 90.0% | IgG 96.2% IgM 98.7% | <https://www.fda.gov/media/141550/download> |
| BioCheck, Inc. | BioCheck SARS-CoV-2 IgM Antibody Test Kit | IgM | CLIA | S1 | 95.45% | 97.20% | <https://www.fda.gov/media/142002/download> |
| BioCheck, Inc. | BioCheck SARS-CoV-2 IgG Antibody Test Kit | IgG | CLIA | S1 | 99.09% | 100% | <https://www.fda.gov/media/142006/download> |
| BioCheck, Inc. | BioCheck SARS-CoV-2 IgG and IgM Combo Test | IgM and IgG | CLIA | S1 | IgG 100% IgM 89% | IgG 100% IgM 97.2% | <https://www.fda.gov/media/141251/download> |
| Biohit Healthcare (Hefei) Co. Ltd. | Biohit SARS-CoV-2 IgM/IgG Antibody Test Kit | IgM and IgG | LFA | N | 96.70% | 95% | <https://www.fda.gov/media/139283/download> |
| bioMérieux SA | VIDAS SARS-CoV-2 IgM | IgM | ELFA | — | 100% | 99.9% | <https://www.fda.gov/media/140933/download> |
| bioMérieux SA | VIDAS SARS-CoV-2 IgG | IgG | ELFA | — | 100% | 99.40% | <https://www.fda.gov/media/140937/download> |
| Bio-Rad Laboratories, Inc. | Platelia SARS-CoV-2 Total Ab assay | Total Antibody | ELISA | N | 92.20% | 99.60% | <https://www.fda.gov/media/137493/download> |
| Cellex Inc. | qSARS-CoV-2 IgG/IgM Rapid Test | IgM and IgG | LFA | N and S | 93.80% | 95.60% | <https://www.fda.gov/media/136625/download> |
| DiaSorin Inc. | LIAISON SARS-CoV-2 S1/S2 IgG | IgG | CLIA | S | 97.60% | 99.30% | <https://www.fda.gov/media/137359/download> |
| DiaSorin, Inc. | DiaSorin LIAISON SARS-CoV-2 IgM Assay | IgM | CLIA | RBD | 92.60% | 99.30% | <https://www.fda.gov/media/142552/download> |
| Diazyme Laboratories, Inc. | Diazyme DZ-Lite SARS-CoV-2 IgG CLIA Kit | IgG | CLIA | — | 100% | 97.40% | <https://www.fda.gov/media/139865/download> |
| Diazyme Laboratories, Inc. | Diazyme DZ-Lite SARS-CoV-2 IgM CLIA Kit | IgM | CLIA | — | 94.40% | 98.30% | <https://www.fda.gov/media/141255/download> |
| Emory Medical Laboratories | SARS-CoV-2 RBD IgG test | IgG | ELISA | RBD | 100% | 96.40% | <https://www.fda.gov/media/139053/download> |
| EUROIMMUN US Inc. | Anti-SARS-CoV-2 ELISA (IgG) | IgG | ELISA | S1 | 90% | 100% | <https://www.fda.gov/media/137609/download> |
| Genalyte, Inc. | Maverick SARS-CoV-2 Multi-Antigen Serology Panel v2 | Total Antibody | Photonic ring immunoassay | Multi-Antigenic sites | 96.13% | 97.68% | <https://www.fda.gov/media/142915/download> |
| GenScript USA Inc. | cPass SARS-CoV-2 Neutralization Antibody Detection Kit 11/06/2020 | Total Neutralizing Antibodies | ELISA | S1 RBD | 96.70% | 94% | <https://www.fda.gov/media/143583/download> |
| Hangzhou Biotest Biotech Co., Ltd. | RightSign COVID-19 IgG/IgM Rapid Test Cassette | IgM and IgG | LFA | S | IgG 100%. IgM 85% | IgG 98% IgM 96% | <https://www.fda.gov/media/139410/download> |
| Hangzhou Laihe Biotech Co., Ltd. | LYHER Novel Coronavirus (2019-nCoV) IgM/IgG Antibody Combo Test Kit (Colloidal Gold) | IgM and IgG | LFA | S1 | IgG 98.5% IgM 99.25% | IgG 99.43% IgM 99.43% | <https://www.fda.gov/media/139410/download> |
| Healgen Scientific LLC | COVID-19 IgG/IgM Rapid Test Cassette (Whole Blood/Serum/Plasma) | IgM and IgG | LFA | S | 100% | 97.50% | <https://www.fda.gov/media/138438/download> |
| Immunodiagnostic Systems Ltd. | IDS SARS-CoV-2 IgG | IgG | CLIA | N and S | 97.60% | 99.60% | <https://www.fda.gov/media/145944/download> |
| InBios International, Inc. | SCoV-2 Detect IgM ELISA | IgM | ELISA | — | 91.89% | 98.95% | <https://www.fda.gov/media/139730/download> |
| InBios International, Inc. | SCoV-2 Detect IgG ELISA | IgG | ELISA | — | 97.78% | 98.95% | <https://www.fda.gov/media/138810/download> |
| Innovita (Tangshan) Biological Technology Co., Ltd. | Innovita 2019-nCoV Ab Test (Colloidal Gold) | IgM and IgG | LFA | N and S1 | IgG 93.3% IgM 93.3% | IgG 98.8% IgM 98.8% | <https://www.fda.gov/media/144071/download> |
| Inova Diagnostics, Inc. | QUANTA Flash SARS-CoV-2 IgG | IgG | CLIA | N and S | 100.00% | 99.90% | <https://www.fda.gov/media/147808/download> |
| Jiangsu Well Biotech Co., Ltd. | Orawell IgM/IgG Rapid Test | IgM and IgG | LFA | RBD | 97.14% | 100% | <https://www.fda.gov/media/142422/download> |
| Kantaro Biosciences, LLC | COVID-SeroKlir, Kantaro Semi-Quantitative SARS-CoV-2 IgG Antibody Kit | IgG | ELISA | RBD | 98.78% | 99.60% | <https://www.fda.gov/media/144010/download> |
| Luminex Corporation | xMAP SARS-CoV-2 Multi-Antigen IgG Assay | IgG | FMIA | N, RBD and S1 | ＞96% | ＞99% | <https://www.fda.gov/media/140256/download> |
| Megna Health, Inc. | Rapid COVID-19 IgM/IgG Combo Test Kit | IgM and IgG | LFA | N | IgG 100% IgM 83.3% | IgG 97.5% IgM 97.5% | <https://www.fda.gov/media/140297/download> |
| Mount Sinai Laboratory | COVID-19 ELISA IgG Antibody Test | IgG | ELISA | S | 92.50% | 100% | <https://www.fda.gov/media/137029/download> |
| NanoEntek America, Inc. | FREND COVID-19 total Ab | Total Antibody | FIA | N | 100% | 99.20% | <https://www.fda.gov/media/142557/download> |
| Nirmidas Biotech, Inc. | MidaSpot COVID-19 Antibody Combo Detection Kit | IgM and IgG | LFA | RBD | IgG 100% IgM 100% | IgG 100% IgM 97.7% | <https://www.fda.gov/media/144877/download> |
| Nirmidas Biotech, Inc. | Nirmidas COVID-19 (SARS-CoV-2) IgM/IgG Antibody Detection Kit | IgM and IgG | LFA | S1 and RBD | IgG 87.9% IgM 93.1% | IgG 100% IgM 97.9% | <https://www.fda.gov/media/142561/download> |
| Ortho Clinical Diagnostics, Inc. | VITROS Immunodiagnostic Products Anti-SARS-CoV-2 Total Reagent Pack | Total Antibody | CLIA | S | 100% | 100% | <https://www.fda.gov/media/136967/download> |
| Ortho-Clinical Diagnostics, Inc. | VITROS Immunodiagnostic Products Anti-SARS-CoV-2 IgG Reagent Pack | IgG | CLIA | S | 90% | 100% | <https://www.fda.gov/media/137363/download> |
| Phadia AB | EliA SARS-CoV-2-Sp1 IgG Test | IgG | ELFA | S1 | 97.6 % | 99.4% | <https://www.fda.gov/media/145089/download> |
| Quansys Biosciences, Inc. | Q-Plex SARS-CoV-2 Human IgG (4 Plex)10/28/2020 | IgG | CLIA | S | 95.20% | 99.70% | <https://www.fda.gov/media/143452/download> |
| Quanterix Corporation | Simoa Semi-Quantitative SARS-CoV-2 IgG Antibody Test | IgG | ELISA | S | 100.00% | 99.19% | <https://www.fda.gov/media/144764/download> |
| Quotient Suisse SA | MosaiQ COVID-19 Antibody Magazine | Total Antibody | Photometric immunoassay | S | 93% | 99.80% | <https://www.fda.gov/media/142486/download> |
| Roche Diagnostics | Elecsys Anti-SARS-CoV-2 S | Total Antibody | ECLIA | RBD | 96.60% | 99.98% | <https://www.fda.gov/media/144037/download> |
| Roche Diagnostics | Elecsys Anti-SARS-CoV-2 | Total Antibody | ECLIA | Np | 100% | 99.80% | <https://www.fda.gov/media/137605/download> |
| Salofa Oy | Sienna-Clarity COVIBLOCK COVID-19 IgG/IgM Rapid Test Cassette | IgM and IgG | LFA | RBD | IgG 93.3% IgM 90% | IgG 98.8% IgM 100% | <https://www.fda.gov/media/140082/download> |
| Shenzhen New Industries Biomedical Engineering Co., Ltd. | MAGLUMI 2019-nCoV IgM/IgG | IgM and IgG | CLIA | N and S | 93.94% | 98.67% | <https://www.fda.gov/media/142233/download> |
| Siemens Healthcare Diagnostics Inc. | Dimension EXL SARS‑CoV‑2 IgG (CV2G) | IgG | CLIA | S1 RBD | 100% | 100% | <https://www.fda.gov/media/145015/download> |
| Siemens Healthcare Diagnostics Inc. | Dimension Vista SARS‑CoV‑2 IgG (COV2G) | IgG | CLIA | S1 RBD | 100% | 100% | <https://www.fda.gov/media/145020/download> |
| Siemens Healthcare Diagnostics Inc. | Atellica IM SARS-CoV-2 IgG (sCOVG) | IgG | CLIA | S1 RBD | 95.58% | 99.90% | <https://www.fda.gov/media/146931/download> |
| Siemens Healthcare Diagnostics Inc. | Dimension Vista SARS-CoV-2 Total antibody assay (COV2T) | Total Antibody | CLIA | S1 RBD | 100% | 99.80% | <https://www.fda.gov/media/138753/download> |
| Siemens Healthcare Diagnostics Inc. | Dimension EXL SARS-CoV-2 Total antibody assay (CV2T) | Total Antibody | CLIA | S1 RBD | 100% | 99.87% | <https://www.fda.gov/media/138757/download> |
| Siemens Healthcare Diagnostics Inc. | Atellica IM SARS-CoV-2 IgG (COV2G) | IgG | CLIA | S1 RBD | 100% | 99.95% | <https://www.fda.gov/media/140699/download> |
| Siemens Healthcare Diagnostics Inc. | ADVIA Centaur SARS-CoV-2 IgG (COV2G) | IgG | CLIA | S1 RBD | 100% | 99.89% | <https://www.fda.gov/media/140704/download> |
| Siemens Healthcare Diagnostics Inc. | Atellica IM SARS-CoV-2 Total (COV2T) | Total Antibody | CLIA | S1 RBD | 100% | 99.82% | <https://www.fda.gov/media/138442/download> |
| Siemens Healthcare Diagnostics Inc. | ADVIA Centaur SARS-CoV-2 Total (COV2T) | Total Antibody | CLIA | S1 RBD | 100% | 99.81% | <https://www.fda.gov/media/138446/download> |
| Sugentech, Inc. | SGTi-flex COVID-19 IgG | IgG | LFA | N and S RBD | 96.70% | 100% | <https://www.fda.gov/media/141891/download> |
| Symbiotica, Inc. | COVID-19 Self-Collected Antibody Test System | IgG | ELISA | — | 100% | 98.0% | <https://www.fda.gov/media/147369/download> |
| TBG Biotechnology Corp. | TBG SARS-CoV-2 IgG / IgM Rapid Test Kit | IgM and IgG | LFA | N and S | IgG 93.3% IgM 93.3% | IgG 96.2% IgM 95.0% | <https://www.fda.gov/media/141773/download> |
| Thermo Fisher Scientific | OmniPATH COVID-19 Total Antibody ELISA Test | Total Antibody | ELISA | — | 96.70% | 97.50% | <https://www.fda.gov/media/142700/download> |
| United Biomedical, Inc. | UBI SARS-CoV-2 ELISA | IgG | ELISA | M, N and S | 89.70% | 100% | <https://www.fda.gov/media/145277/download> |
| University of Arizona Genetics Core for Clinical Services | COVID-19 ELISA pan-Ig Antibody Test | Total Antibody | ELISA | RBD or S2 | 97.50% | 93.30% | <https://www.fda.gov/media/141777/download> |
| Vibrant America Clinical Labs | Vibrant COVID-19 Ab Assay | IgM and IgG | CLIA | S1, RBD, S2, and N | 98.10% | 98.60% | <https://www.fda.gov/media/138629/download> |
| Wadsworth Center, New York State Department of Health | New York SARS-CoV Microsphere Immunoassay for Antibody Detection | Total Antibody | FMIA | N | 88% | 98.80% | <https://www.fda.gov/media/137541/download> |
| Xiamen Biotime Biotechnology Co., Ltd. | BIOTIME SARS-CoV-2 IgG/IgM Rapid Qualitative Test | IgM and IgG | LFA | — | 100% | 96.20% | <https://www.fda.gov/media/140443/download> |
| ZEUS Scientific, Inc. | ZEUS ELISA SARS-CoV-2 IgG Test System | IgG | ELISA | S1 RBD | 93.30% | 100.00% | <https://www.fda.gov/media/142809/download> |
|  |  |  |  |  |  |  |  |

**Abbreviations:** CLIA = Chemiluminescence Immunoassay, CMIA = Chemiluminescent microparticle immunoassay, ECLIA= Electrochemiluminescence Immunoassay, ELFA=Enzyme Linked Fluorescent Assay, ELISA=Enzyme Linked Immunosorbent Assay, FIA= Fluorescence immunoassay, FMIA= Fluorescent-microbead immunoassay, LFA= Lateral Flow Assay.
